# KRAS mutant colorectal cancer gene signatures identified angiotensin II receptor blockers as potential therapies

**DOI:** 10.18632/oncotarget.13884

**Published:** 2016-12-10

**Authors:** Qing Wen, Philip D. Dunne, Paul G. O’Reilly, Gerald Li, Anthony J. Bjourson, Darragh G. McArt, Peter W. Hamilton, Shu-Dong Zhang

**Affiliations:** ^1^ Centre for Cancer Research and Cell Biology, Queen's University Belfast, UK; ^2^ Northern Ireland Centre for Stratified Medicine, Biomedical Sciences Research Institute, Ulster University, C-TRIC, Londonderry, UK

**Keywords:** connectivity mapping, differentially expressed genes, colorectal cancer, KRAS mutation, FDA approved drugs

## Abstract

Colorectal cancer (CRC) is a life-threatening disease with high prevalence and mortality worldwide. The KRAS oncogene is mutated in approximately 40% of CRCs. While antibody based EGFR inhibitors (cetuximab and panitumumab) represent a major treatment strategy for advanced KRAS wild type (KRAS-WT) CRCs, there still remains no effective therapeutic course for advanced KRAS mutant (KRAS-MT) CRC patients.

In this study, we employed a novel and comprehensive approach of gene expression connectivity mapping (GECM) to identify candidate compounds to target KRAS-MT tumors. We first created a combined KRAS-MT gene signature with 248 ranked significant genes using 677 CRC clinical samples. A series of 248 sub-signatures was then created containing an increasing number of the top ranked genes. As an input to GECM analysis, each sub-signature was translated into a statistically significant therapeutic drugs list, which was finally combined to obtain a single list of significant drugs.

We identify four antihypertensive angiotensin II receptor blockers (ARBs) within the top 30 significant drugs indicating that these drugs have a mechanism of action that can alter the KRAS-MT CRC oncogenic signaling. A hypergeometric test (*p-value* = 6.57 × 10^−6^) confirmed that ARBs are significantly enriched in our results. These findings support the hypothesis that ARB antihypertensive drugs may directly block KRAS signaling resulting in improvement in patient outcome or, through a reversion to a KRAS wild-type phenotype, improve the response to anti-EGFR treatment. Antihypertensive angiotensin II receptor blockers are therefore worth further investigation as potential therapeutic candidates in this difficult category of advanced colorectal cancers.

## INTRODUCTION

Colorectal cancer (CRC) is a life-threating disease with high prevalence and mortality worldwide. According to the American Cancer Society, an estimated 134,490 new CRC cases were diagnosed and 49,190 colon and rectum cancer deaths occurred in the U.S. in 2016, making CRC the 4th highest in incidence and the 2nd highest in mortality of malignant cancers [1]. In the U.K., it has been projected that by 2020, there will be 340,000 patients living with CRC placing it third among malignant cancer types after breast and prostate cancer [2]. CRC is the second most common cause of cancer death in the U.K., behind lung cancer [3].

The steps involved in CRC development have been shown to align with the accumulation of genetic mutations, resulting in more aggressive disease at later stages, highlighting early detection and intervention as a key step in reducing the risk of cancer progression and death [3]. With advances in genomics technology the understanding and knowledge of CRC at a molecular level is being updated by evaluating gene expression profiles of cancerous and healthy samples. The identification of genes or proteins associated with CRC development as potential biomarkers not only assists the early detection of CRC, but also helps to shed light on the mechanisms of disease development and progression [4]. Although considerable research effort has been devoted to the discovery of CRC molecular markers and drugs, many published prognostic and predictive markers are inconsistent [5, 6]. The Kirsten Ras (KRAS) gene, a prominent member of the RAS family, however was the first predictive marker approved by the U.S. Food and Drug Administration (FDA) for treatment of CRC and represents a unique biomarker for epidermal growth factor receptor (EGFR)-targeted therapy currently used in clinical practice [7].

RAS proteins are a family of related proteins sharing very similar GDP/GTP binding domains, and play important roles in cell growth, differentiation and survival. Characterized by overexpression, activating mutations or upstream activation, dysregulation of RAS proteins occurs frequently in human cancers. While there are many members in this family, the most notable ones are HRAS, KRAS and NRAS because of their involvement in human cancers [8]. KRAS is found to be mutated in a number of human cancers, such as CRC, pancreatic and lung cancer. Mutations in the KRAS oncogene are common, with a prevalence of approximately 40% in CRCs [9]. KRAS has become a key target for therapeutic drug development, methods to suppress KRAS activity, particularly by disrupting KRAS signaling, have been explored in numerous studies [10–14]. While there has been substantial effort afforded to KRAS directed drug development, currently there are no approved drugs to inhibit KRAS for clinical use and KRAS mutation has long been considered “undruggable” [15].

Gene Expression Connectivity Mapping (GECM) provides an innovative approach in the discovery of new candidate compounds from previously FDA approved drugs, which may provide new insights into possible treatment strategies for KRAS mutant (KRAS-MT) colorectal cancer patients. Connectivity mapping was first proposed and developed by Lamb and colleagues at the Broad Institute in 2006 [16]. Using a reference database, a query signature and a matching algorithm, it creates a link between genes, drugs and a biological condition [17]. The reference database comprises a large collection of data on the differential gene expression effects of small molecule compounds applied to cell lines. A query gene signature is a list of genes selected by the researcher based on experimental results, which represents a biological condition such as a disease. To measure the similarity between the query signature and the differential expression profiles of drugs in the reference database, a non-parametric pattern matching method is used to calculate a connection score for each compound in the reference database indicating its affinity to this biological condition. If the gene expression profile of a compound in the reference database has the closest pattern match but regulates in the opposite direction of the query signature, the drug receives the most negative score, implying it has the potential to reverse the corresponding biological condition [16, 17]. Since the initial concept was introduced, research has been done to improve the capabilities of connectivity mapping [18–22]. The sscMap represented some major efforts to refine the scoring scheme and offered noteworthy improvements on the connectivity mapping procedure [19]. Utilizing the gene expression profiling data released by the Broad Institute as the reference database, sscMap rebuilt a refined framework of connectivity mapping to control false discoveries, through calculations of *p*-value at individual treatment instance level [18]. Connectivity mapping is a powerful research tool to discover novel drugs for disease and particularly for repurposing existing drugs [23–26]. While the first version of connectivity map (build 01) included 164 small molecules and 564 gene expression profiles, the Broad Institute recently released the Library of Integrated Cellular Signatures (LINCS) transcriptomics data via the LINCS Cloud, which contains over 1.3 million reference profiles including those for over 20,000 small molecules applied to 77 cell lines [27]. While the enormous expansion of data makes this research tool more powerful and appealing, screening the vast number of significant drugs in the analysis can take considerable time. As many of the compounds in the reference database are FDA approved, we utilize a novel *in silico* method to pre-screen the drug profiles to only include FDA approved drugs as the core collection of reference compounds for our main GECM analyses. This means that any positive hit has been deemed safe as a therapeutic option and could potentially skip phase I and enter directly to the phase II clinical trials much quicker than non-FDA approved compounds. This could save enormous amounts of time and effort in the process of drug discovery and development [28–30].

In the current paper, we propose a novel and comprehensive procedure for connectivity mapping from the creation of a robust query signature to establishing new connections between the most significant gene sets and the most significant drugs in the database. Using KRAS-MT and KRAS wild type (KRAS-WT) expression profiles to generate a robust query gene signature for comparison against a subset of the LINCS data containing 1354 FDA approved drugs, this study aims to identify new compounds for the treatment of KRAS-MT CRCs by making them more amenable to the EGFR-targeted therapies that have been effective against KRAS-WT tumors.

## RESULTS

### Significant genes and their pathway analysis

To create query gene signatures for connectivity mapping, CRC datasets GSE35896, GSE39084 and GSE39582 containing microarray expression raw data and associated KRAS mutation status were selected from Gene Expression Omnibus (GEO). A total of 677 colorectal cancer samples were used for our analysis, from which a significant gene list of 248 probes was generated by combining significant genes from the results of differential analysis of the selected datasets (see Supplementary Table S1 for the full list of 248 significant gene probes).

**Figure 1:**
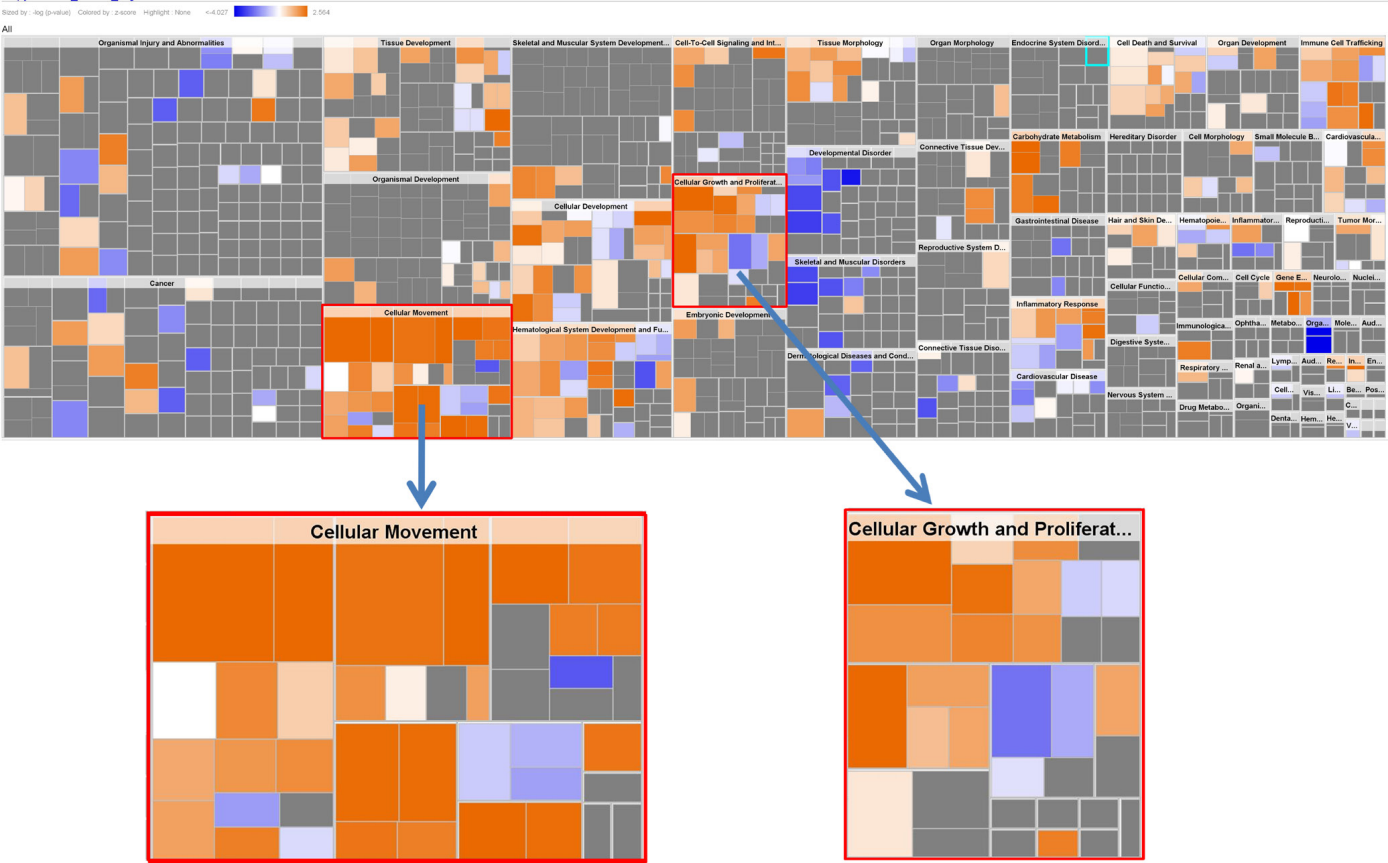
IPA Downstream Effects Analysis: Biological activities associated with the CRC KRAS-MT gene signature Each individual square is a particular biological function or process. Orange squares indicate increases and blue squares decreases in the functions. A gray square means no predicted change in that particular function. In this figure, cellular movement and cellular growth/proliferation are prominently shown to have increased activities.

**Figure 2:**
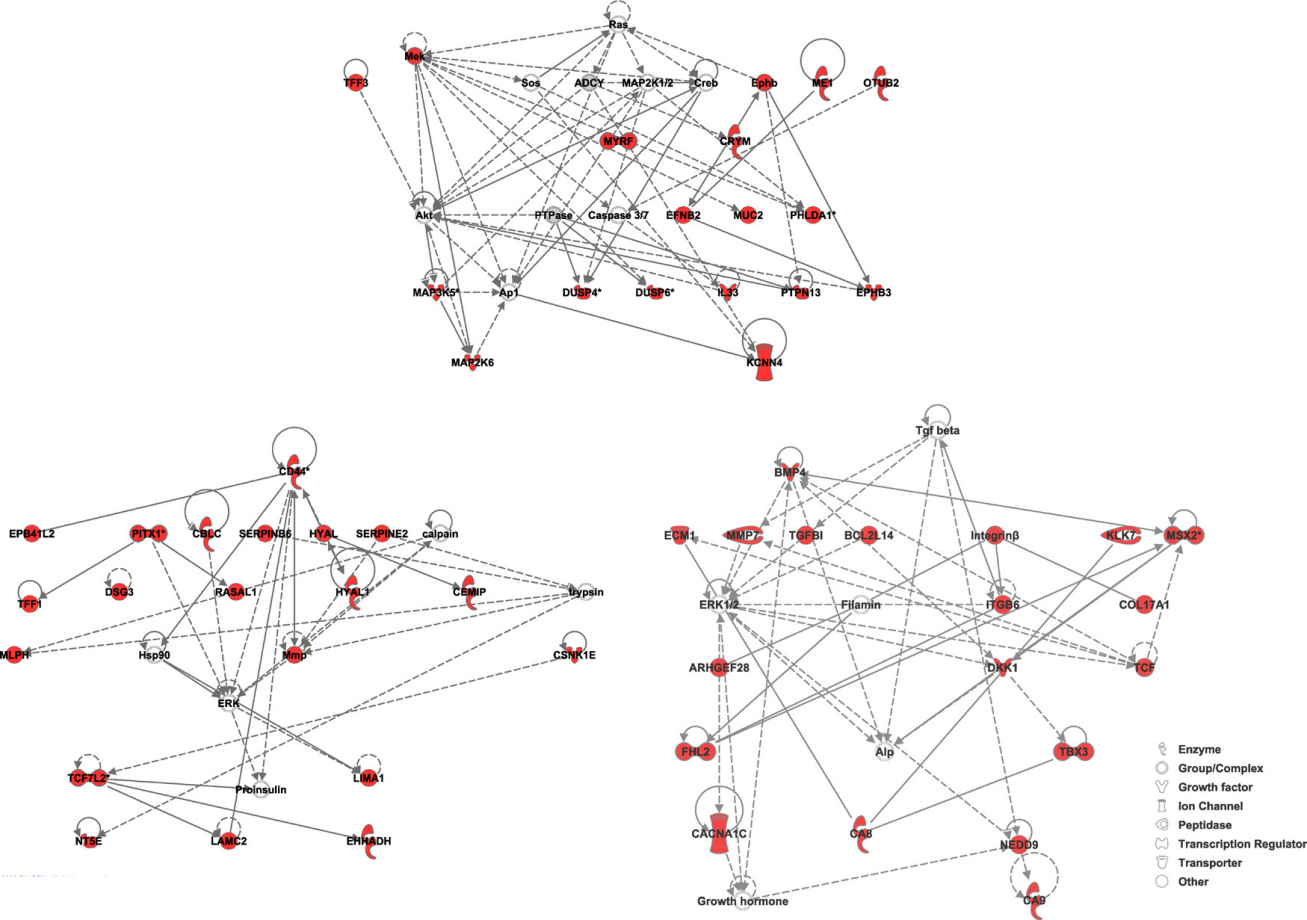
IPA Network Analysis using the KRAS-MT gene signature We observe that the MAPK/ERK pathway is highlighted in the 3 highest scoring networks. Members of the gene signature are highlighted in red.

KEGG (Kyoto Encyclopedia of Genes and Genomes) pathway analysis was carried out to investigate the relationship of significant genes to known pathways [31]. Table 1 shows 17 significant pathways (*p* < 0.05) with PPAR signaling pathway, Wnt signaling pathway and MAPK signaling pathway being highly enriched. Using QIAGEN's Ingenuity Pathway Analysis to compare KRAS-MT and KRAS-WT profiles, our list of 248 differentially expressed probes, representing 201 unique annotated genes, showed upregulation in 133 genes and downregulation in 68 genes (see Supplementary Table S2). In line with the known role of activating mutations in KRAS, cell migration/movement and cell growth/proliferation were predicted to be highly activated in our combined signature (Figure 1). The upstream regulators of these functions were predicted to involve the growth factors TGFB1, EGF, HGF and IGF1, which have known roles in activating these cancer progression pathways (Supplementary Table S2). Using network analysis to further interrogate the biological signaling in our combined KRAS-MT signature, we observe that the MAPK/ERK pathway is highlighted in each of the 3 highest scoring networks (Figure 2) giving us confidence that the underlying biology represented by the combined signature is representative of activation of the KRAS pathway.

**Table 1:** Top KEGG pathways associated with the KRAS-MT CRC gene signature

PathwayID	Pathway Name	P value	Pathway Members	Overlaps
hsa05412	Arrhythmogenic right ventricular cardiomyopathy	0.0025	67	6
hsa00910	Nitrogen metabolism	0.0039	16	3
hsa00760	Nicotinate and nicotinamide metabolism	0.0056	18	3
hsa03320	PPAR signaling pathway	0.0077	60	5
hsa04610	Complement and coagulation cascades	0.0114	66	5
hsa04310	Wnt signaling pathway	0.0129	122	7
hsa00920	Sulfur metabolism	0.0138	9	2
hsa05217	Basal cell carcinoma	0.0144	46	4
hsa04520	Adherens junction	0.0152	71	5
hsa05146	Amoebiasis	0.0194	103	6
hsa04010	MAPK signaling pathway	0.0211	231	10
hsa05216	Thyroid cancer	0.0211	29	3
hsa04142	Lysosome	0.0238	108	6
hsa00450	Selenocompound metabolism	0.0242	12	2
hsa00350	Tyrosine metabolism	0.0274	32	3
hsa05215	Prostate cancer	0.0306	85	5
hsa04916	Melanogenesis	0.0362	89	5

PPAR signaling, Wnt signaling, and MAPK signaling are among the top enriched pathways that are known to be highly relevant to current biological context. Pathway IDs and names are extracted from the KEGG database; Pathway members give the number of genes on the Affymetrix U133A microarray platform that are known to be associated with the particular pathway. *P*-value was obtained from a hypergeometric test assessing the statistical significance of the number of overlap genes between the signature and the pathway members present on the microarray.

### Significant drugs

Having firmly established the quality and biological relevance of our gene list, GECM was performed in a comprehensive manner. From our list of 248 significant probes, a series of 248 sub-signatures was created and used as inputs to run connectivity mapping. This series of GECM runs returned 248 separate lists of significant drug candidates. After re-combining and prioritizing the drug result, 286 drugs are promising candidates to reverse/alter the KRAS-MT phenotype. Table 2 shows the top drugs with absolute mean score > 0.50 in the result list.

**Table 2:** Top drugs with absolute mean score > 0.50 in the result list

Drug Name	Replicates	Sumscore	Meanscore	absMeanscore	Rank
Trametinib; JTP-74057; GSK1120212	133	–241.5	–0.974	0.974	1
D-cycloserine	93	–220.3	–0.888	0.888	2
GW-572016; Lapatinib; Tykerb	178	–220.0	–0.887	0.887	3
Rizatriptan	72	–213.2	–0.860	0.860	4
SA59353	24	–192.9	–0.778	0.778	5
Selegiline	37	–192.5	–0.776	0.776	6
Bosentan	55	–191.2	–0.771	0.771	7
Tolterodine	35	–188.9	–0.762	0.762	8
Irinotecan	36	–187.0	–0.754	0.754	9
Budesonide	85	–179.9	–0.725	0.725	10
Ponatinib; AP24534	132	–178.8	–0.721	0.721	11
Eprosartan	45	–177.1	–0.714	0.714	12
Donepezil	73	–175.5	–0.707	0.707	13
Tetrahydrobiopterin	45	–172.8	–0.697	0.697	14
Metaproterenol	44	–171.9	–0.693	0.693	15
Irbesartan	120	–163.0	–0.657	0.657	16
Brimonidine	81	–157.3	–0.634	0.634	17
Caffeine	116	–146.5	–0.591	0.591	18
Meropenem	81	–145.2	–0.586	0.586	19
Lidocaine	73	–133.3	–0.537	0.537	20
Granisetron	10	–131.1	–0.529	0.529	21
Losartan	70	–129.0	–0.520	0.520	22
Nitrazepam	37	–124.5	–0.502	0.502	23

The drugs’ scores across 248 lists were summed and their mean score calculated. This table lists the drug in descending order of absolute mean score. Replicates give the number of reference profiles in the LINCS database for that particular drug.

Trametinib (JTP-74057, GSK1120212) is the drug with the highest absolute overall score in the significant drug list. The sum score of trametinib across the 248 drug lists is –241.5 out of a possible maximum of (+/−)248, and the mean score is –0.974. D-cycloserine and lapatinib are the second and third overall-ranked drugs, which have sum score of –220.3 and –220.0, and mean score of -0.888 and –0.887, respectively (Table 2).

A number of cancer related drugs are on the significant drugs list including some already used for the treatment of CRC. Top scoring drug trametinib is used for the treatment of unresectable or metastatic melanoma with BRAF V600E or V600K mutations. The third-ranked drug lapatinib is used as a treatment for solid tumors such as breast and lung cancer. Irinotecan (ranked 9) has been used as a first-line therapy to treat metastatic colorectal cancer. Ponatini (ranked 11) was approved by the FDA in 2012 for the treatment of chronic myeloid leukemia [32]. Trametinib, lapatinib and ponatini are known signal transduction inhibitors. Currently used colorectal cancer chemotherapy drugs include etoposide (ranked 67) and 5-Fluorouracil (ranked 262) [32, 33].

Interestingly, we found that antihypertensive drugs are highly represented on our significant drugs list. There are 7 antihypertensive drugs among the top 100 drugs, including eprosartan (ranked 12), irbesartan (16), losartan (22), olmesartan (28), benazepril (34), fenoldopam (64), labetalol (84). Eprosartan, irbesartan, losartan, and olmesartan are angiotensin II receptor blockers (ARBs), which are within the top 30 of significant drugs. Table 3 shows four ARB drugs in the result list. There are a total of 8 FDA approved ARB drugs being used in the U.S. Apart from azilsartan, seven ARB drugs (candesartan, eprosartan, irbesartan, losartan, olmesartan, telmisartan and valsartan) are included in the LINCS data among the 1354 FDA drugs. Four of them were within the top 30 significant drugs in the results. A hypergeometric test returned a *p*-value of 6.57 × 10^−6^ suggesting ARB drugs are highly enriched among the top drugs. In addition, we found that two EGFR inhibitors, lapatinib and lidocaine, are among the top 30 GECM predicted drugs. As there were five EGFR inhibitors, afatinib, gefitinib, erlotinib, lapatinib, and lidocaine, included in the pool of 1354 FDA drugs, a hypergeometric test returned a *p*-value of 0.0046, indicating the enrichment of EGFR inhibitors among the top 30 drugs is also statistically significant. Of these 5 EGFR inhibitors listed above, afatinib and lapatinib are also ERBB2 inhibitors. And they are the only known ERBB2 inhibitors included in the LINCS 1354 FDA drugs. One of these two, lapatinib made into the top 30 drugs. Similarly, we also performed the hypergeometric test on enrichment of ERBB2 inhibitors and MAP2K1/2 inhibitors, as these were well known targets of the top drug #1 or #3; the results are included in Supplementary Table S3. As can be seen from these results, the enrichment of ARB drugs is far more significant than the others tested. We consequently focused on the ARB drugs as our main findings in this work.

**Table 3:** Antihypertensive angiotensin II receptor blocker (ARB) drugs in our result list

Drug Names	Replicates	Indication	Mechanism of action	Rank
Eprosartan	45	Hypertension, diabetic nephropathy, congestive heart failure	angiotensin II receptor blocker (ARB): inhibits the binding of angiotensin II to angiotensin II type 1 (AT1) receptor	12
Irbesartan	120	Hypertension, diabetic nephropathy, congestive heart failure	angiotensin II receptor blocker (ARB): inhibits the binding of angiotensin II to its type 1 (AT1) receptor	16
Losartan	70	Hypertension, diabetic nephropathy, congestive heart failure, myocardial infarction	angiotensin II receptor blocker (ARB): inhibits the binding of angiotensin II to its type 1 (AT1) receptor	22
Olmesartan	37	Hypertension	angiotensin II receptor blocker (ARB): inhibits the binding of angiotensin II to its type 1 (AT1) receptor	28

We have checked the pharmacodynamics of the top drugs in the ranked list. ARB drugs are found to be prominently represented among the top drugs of the list. This is statistically very significant with a *p*-value of 6.57 × 10^−6^ (hypergeometric test).

### Contributive genes for the Antihypertensive drugs

**Figure 3:**
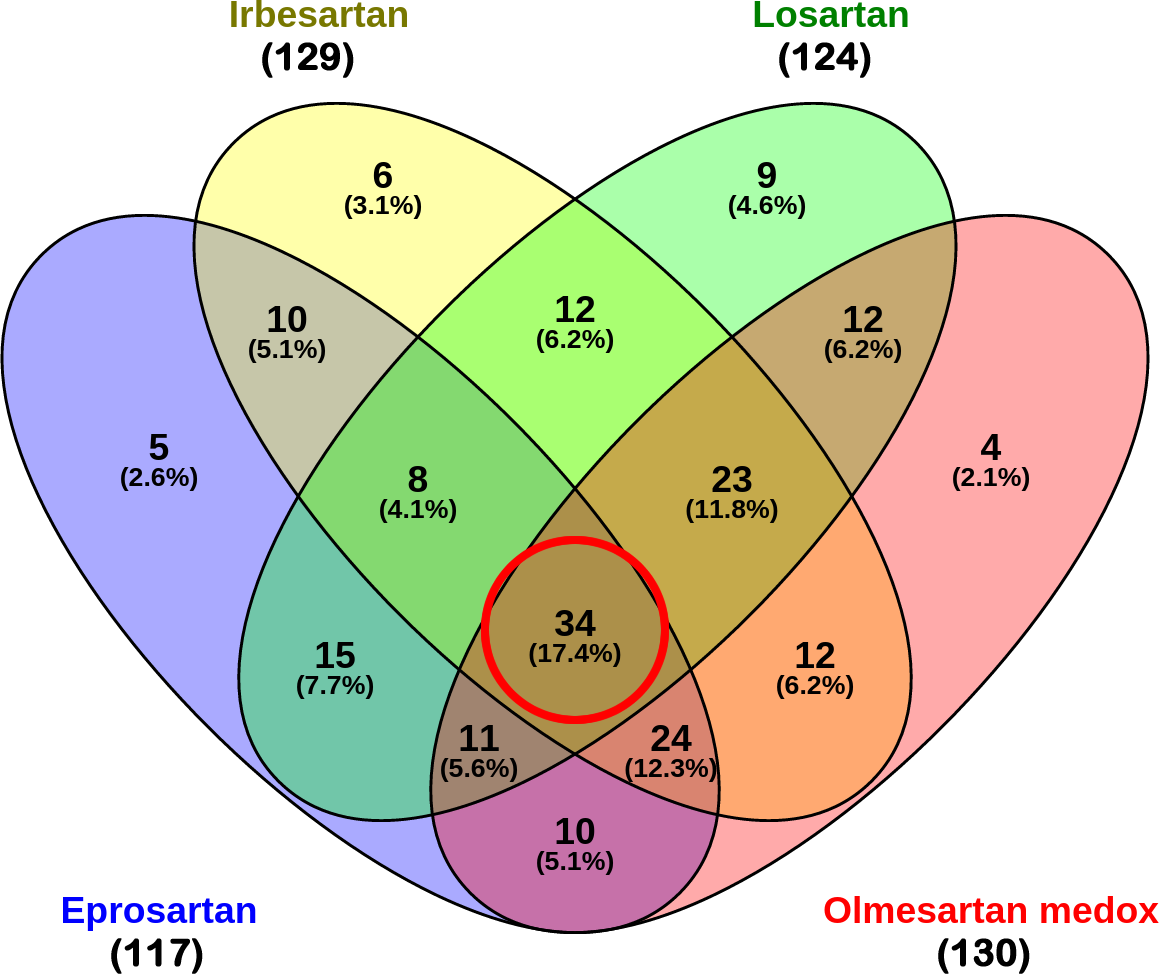
Venn diagram of contributive genes of the four ARB drugs Out of 248 genes, 117, 129, 124 and 130 contributive genes were identified for eprosartan, irbesartan, losartan and olmesartan respectively. There are 34 contributive genes common to all four ARBs, and 66 contributive genes common to 3 out of 4 ARBs.

In order to discern which signature genes contributed to the significant connections between the antihypertensive drugs and the KRAS-MT CRC disease state, we extracted the LINCS reference gene expression profiles for the ARB hypertension drugs detailed in Table 3. In analyzing the contributions of the probes in the signature towards the negative connection scores of these drugs, we obtained lists of 117, 129, 124 and 130 contributive genes for eprosartan, irbesartan, losartan and olmesartan, respectively (see Supplementary Table S4 for these four lists of contributive genes). These gene lists were analyzed using Venny to find the overlap between the four ARB drugs [34]. As can be seen from Figure 3, there are 34 genes common to all four drugs, with a further 66 present in at least three lists out of the four (see Table 4 for details of the common 34 genes).

**Table 4:** The list of 34 contributive genes common to the 4 ARB drugs

Gene Symbol	Description	Regulation
ABHD2	Abhydrolase Domain Containing 2	Up
APOBEC1	Apolipoprotein B mRNA Editing Enzyme, Catalytic Polypeptide 1	Up
BCL2L14	Bcl2-Like 14 (Apoptosis Facilitator)	Up
C3orf52	Chromosome 3 Open Reading Frame 52	Up
CD44	Cd44 Molecule (Indian Blood Group)	Up
CD55	Cd55 Molecule, Decay Accelerating Factor For Complement (Cromer Blood Group)	Up
CKAP4	Cytoskeleton-Associated Protein 4	Up
CTSE	Cathepsin E	Up
DSG3	Desmoglein 3	Up
DUSP4	Dual Specificity Phosphatase 4	Up
EPS8L1	Eps8-Like 1	Up
HGD	Homogentisate 1,2-Dioxygenase	Up
HOXB3	Homeobox B3	Up
HOXB5	Homeobox B5	Up
HOXB6	Homeobox B6	Up
HOXB7	Homeobox B7	Up
HOXB9	Homeobox B9	Up
IL33	Interleukin 33	Up
KIAA1199	Cell Migration Inducing Protein, Hyaluronan Binding [CEMIP]	Up
KRT6B	Keratin 6B	Up
LYZ	Lysozyme	Up
MAP3K5	Mitogen-Activated Protein Kinase Kinase Kinase 5	Up
ME1	Malic Enzyme 1, NADP(+)-Dependent, Cytosolic	Up
MSX2	Msh Homeobox 2	Up
MUC2	Mucin 2, Oligomeric Mucus/Gel-Forming	Up
NAAA	N-Acylethanolamine Acid Amidase	Down
RBMS1	RNA Binding Motif, Single Stranded Interacting Protein 1	Down
REEP1	Receptor Accessory Protein 1	Down
RGNEF	Rho Guanine Nucleotide Exchange Factor (GEF) 28 [ARHGEF28]	Up
SCRN1	Secernin 1	Down
TFF1	Trefoil Factor 1	Up
TFF3	Trefoil Factor 3 (Intestinal)	Up
TRIM16	Tripartite Motif Containing 16	Up
ZBTB10	Zinc Finger And Btb Domain Containing 10	Down

The signed ranks of signature genes in the reference profiles of 4 ARB drugs were analyzed in conjunction with their regulation directions. Genes that make a strong and consistent contribution to the inverse connection scores are selected for each of the 4 ARB drugs. The 4 lists of contributive genes were overlapped and the common set of 34 is itemized here. Their directions of regulation in the signature are indicated in the last column.

**Figure 4:**
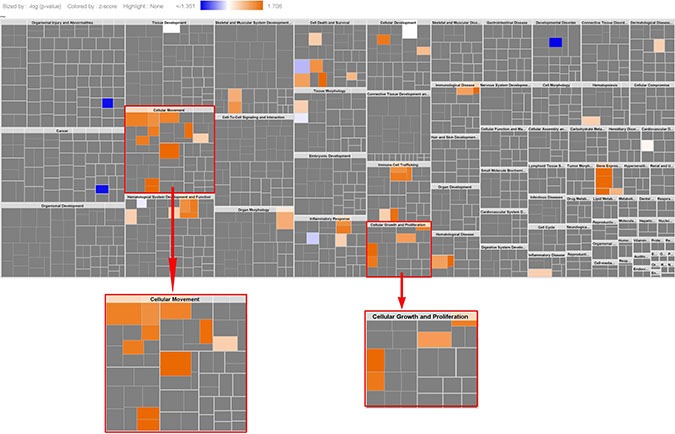
Phenotypical function analysis (34 genes) The results show cell migration, cell growth/proliferation were highly activated (this is same as the activated pathways in the original KRAS-MT CRC signature, see Figure 1), indicating ARBs target cell migration, cell growth/proliferation.

**Figure 5:**
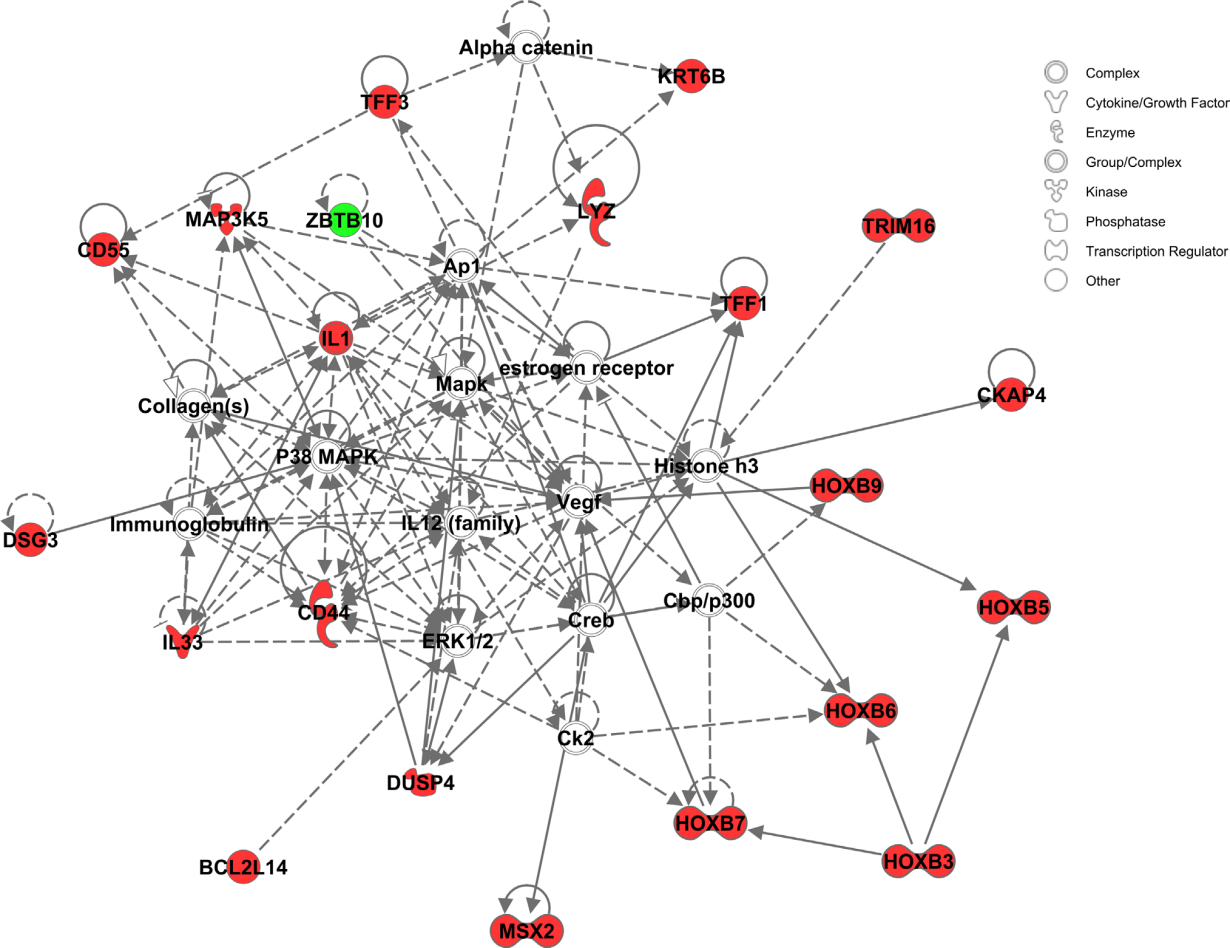
The signaling and interaction network associated with the 34 common contributive genes for the four ARB drugs MAPK/ERK signaling is seen at the heart of this network. Red nodes represent genes up-regulated, green nodes down-regulated, and gray nodes not among the 34 common genes.

Using Ingenuity Pathway Analysis (IPA) we carried out phenotype and network predictions based on the 34 common genes contributing to the four ARB anti-hypertension drugs. The common phenotypic pathway which these drugs are predicted to perturb is cell growth/proliferation, which alongside cell migration was the most activated pathway represented in the KRAS-MT tumor signature (Figure 4). Further detailed analysis of these 34 genes contributing to the anti-hypertension drugs, highlights the MAP Kinase specific nature of their action (Figure 5), underpinning their KRAS-directed mechanism of action.

### Validation of the GECM approach against drug screen sensitivity data from independent studies

To validate the GECM approach proposed in this paper, we conducted an extensive *in silico* validation exercise to demonstrate the power and accuracy of our prediction against drug screen sensitivity data from independent studies. This was possible thanks to several recent studies, which screened large collections of genetically characterized human cancer cell lines against hundreds of compounds [35–39]. For our purpose of validating the GECM approach, we were particularly interested in the cell viability reduction data for our top candidate compounds in colorectal cancer cell lines with and without KRAS mutation. The Seashore-Ludlow study and the Garnett study were chosen for this validation exercise.

The Seashore-Ludlow 2015 study [35] screened 481 compounds, 286 of which were also included in the LINCS compounds collection. This common set of compounds allowed us to validate the prediction of GECM. Using the LINCS reference gene expression profiles for these 286 compounds as a core database, we performed connectivity mapping analysis using the same KRAS-MT gene signature. This gave a list of top compounds that could potentially benefit the KRAS-MT CRCs. Then we examined the sensitivity of all the CRC cell lines in the Seashore-Ludlow study to these top compounds.

We extracted the drug screening data for the cell lines that were labelled as colorectal cancer (CRC), with the term “large intestine carcinoma”. In total, 49 cell lines were identified as CRC. Of these 49 CRC cell lines, 25 were KRAS-WT and 24 were KRAS-MT. We then compared the sensitivity of GECM predicted compounds in the KRAS-MT versus KRAS-WT CRC cell lines. Among the top 20 GECM predicted drugs (with absolute mean score > 0.25), 16 of them turned out to be more sensitive in KRAS-MT CRC cell lines than in KRAS-WT CRC cell lines. Although the differences in sensitivity were relatively small, the accuracy in predicting the direction of action was 80%. Supplementary Table S5 provides the details of these 20 compounds and the corresponding sensitivity data, area under concentration-response-curve (AUC), as obtained from the Seashore-Ludlow study.

Similar to the analysis performed against the Seashore-Ludlow study above, we investigated a second study from which the KRAS mutation status of assayed cell lines can be readily retrieved. The Garnett 2012 study [38] screened 131 compounds in 639 human tumor cell lines with IC50 data provided as Supplementary Information. Of these 131 Garnett compounds, 121 were included in the LINCS compound collection. We thus extracted all the LINCS reference gene expression profiles for these 121 Garnett compounds and used them as a core database for gene expression connectivity mapping with the KRAS-MT gene signature. Again, a list of top candidate drugs predicted to be effective in KRAS-MT CRCs was returned. Following the same approach, we extracted the drug sensitivity data of these top candidate drugs in all the 34 CRC cell lines from the Garnett study. Sixteen of these CRC cell lines were KRAS-WT and 18 were KRAS-MT. One KRAS-WT cell line was a genetic outlier and discarded because it had a copy number > 8, unlike all the other 15 KRAS-WT cell lines. Among the 13 GECM predicted compounds (with absolute mean score > 0.25), 10 of them turned out to be more sensitive in KRAS-MT CRC cell lines than in the KRAS-WT CRC cell lines. In this case, the accuracy of GECM prediction on the drug's direction of action in the CRC cell lines is 77%. Supplementary Table S6 provides the details of these 13 compounds and the corresponding drug screen sensitivity IC50 data as obtained from the Garnett study.

Taking two independent validation data sets together, the 77% ~ 80% agreement in the direction of action between GECM predictions and cell line drug screen experiments is remarkable. It should be noted that no known ARB drugs were screened in either study above, so we could not compare ARBs sensitivity in the cell lines directly. However, the 77% ~ 80% success rate provided strong evidence that the GECM approach described in this paper is working well. This in turn can provide us with high confidence in our findings that ARBs could be potential drugs used in KRAS-MT CRCs.

We note that in the two independent validation datasets described above, the compounds used in the GECM analysis were not limited to FDA approved drugs. Consequently, some non-FDA approved compounds were also returned by GECM as potentially effective in treating KRAS-MT CRCs. Here we only mention the top hit, selumetinib, from the Seashore-Ludlow compound collection, and PD-0325901, from the Garnett compound collection. Both were more sensitive in KRAS-MT than in KRAS-WT CRC cell lines. Selumetinib, in combination with some existing cancer drugs (eg, docetaxel, erlotinib, cetuximab), is being/has been investigated in clinical trials for treating KRAS-MT solid tumors including non-small-cell lung cancer (NSCLC) [40, 41] and CRC [42]. The experimental drug PD-0325901, too, in combination with an approved drug palbociclib, is being investigated in a clinical trial as a possible treatment for cancers with KRAS mutations, particularly for those which started in the lung (https://clinicaltrials.gov/show/NCT02022982). Details of other GECM hits using the Seashore-Ludlow and the Garnett compound collections can be found in the corresponding Supplementary Files for these two validation sets.

## DISCUSSION

To our knowledge this is the first study using clinical data and connectivity mapping to identify candidate compounds for KRAS-MT CRC. We proposed a comprehensive procedure for connectivity mapping with the aims of getting quality signatures and obtaining robust and meaningful drug results.

We performed differential expression analysis on 677 samples across 3 colorectal cancer datasets, from which 248 gene probes were identified as being differentially expressed between KRAS-MT and KRAS-WT. These 248 gene probes were mapped to known pathways to determine if any signaling networks are overrepresented in the gene signature identified. As a result, PPAR signaling pathway, Wnt signaling pathway and MAPK signaling pathway were identified as among the most prominent pathways represented in our KRAS-MT gene signature.

Peroxisome proliferator-activated receptors (PPARs) are nuclear hormone receptors playing essential roles in the regulation of cellular differentiation, development, metabolism and tumorigenesis [43]. There are three known types of PPARs, including alpha, gamma and delta (beta). PPAR gamma is involved in the regulation of numerous genes regulating cell differentiation and apoptosis [44]. As such, PPAR gamma has been implicated in various diseases including cancers [43]. PPARs can either be tumor suppressors or accelerators (oncogenes), and are therefore potential candidates as drug targets for cancer prevention and treatment [45]. PPAR gamma activators show promise as a future cancer therapeutic [46]. The growth and differentiation of colon cancer cells can be modulated through PPAR gamma where loss-of-function mutations in PPAR gamma have been shown to be associated with colon cancer, suggesting that activation of this receptor might have an anticancer effect in this disease [47, 48].

Wnt signaling pathways regulate cell fate determination, cell migration, cell polarity, neural patterning and organogenesis, and play a critical role in embryonic development [49]. Wnt signaling pathways include the canonical Wnt pathway, the non-canonical planar cell polarity pathway and the non-canonical Wnt/calcium pathway. Wnt signaling has had an association with cancer since its initial discovery. Research found that mutation of the adenomatous polyposis coli gene (APC), which in turn activates the Wnt signaling pathway, is a vital event in the development of colon cancer. Non-canonical Wnt and other distinct pathways in the tumor micro-environment interact with the canonical Wnt pathway and influence colon cancer progression. These non-APC aspects are considered linked to the development of potential treatment for colorectal cancer [50].

Mitogen-activated protein kinase (MAPK, also known as ERK) regulates various cellular functions, including proliferation, gene expression, differentiation, mitosis, cell survival, and apoptosis [51]. A defect in the MAPK/ERK pathways may lead to uncontrolled growth of cells, which is necessary for the development of all cancers. In cancer, RAS and BRAF are among the most frequently mutated members of this extracellular signal-regulated kinase pathway. Therefore, members of MAPK/ERK pathways, particularly RAS and BRAF are potential drug targets, the inhibition of which is a strategy for developing cancer treatment.

The results from KEGG Pathway analysis indicate that the identified significant genes are strongly correlated to PPAR, Wnt, MAPK signaling pathways. These pathways are clearly involved in colorectal cancer progression and development, and are well recognized as potential therapeutic targets. This also demonstrated that our results from differential analysis provided highly accurate gene signatures for KRAS-MT colorectal cancers.

GECM identified 286 significant drugs. A number of colon cancer specific therapeutic agents were retrieved from the drug list providing evidence of credibility in the approach. For example, irinotecan, etoposide and 5-fluorouracil were retrieved as drugs relevant to the CRC signature. The top three significant drugs were trametinib, D-cycloserine and lapatinib, two of which are known cancer therapies and one is a novel discovery.

Trametinib is a MEK inhibitor with anti-cancer activity, as an FDA approved cancer drug used for the treatment of BRAF mutant melanoma. Trametinib decreases cell proliferation and increases cell apoptosis. Research has found that trametinib exhibits a synergistic effect when combined with 5-fluorouracil, oxaliplatin or SN-38; these findings suggest that trametinib is a useful therapeutic drug for colorectal cancer [52], again validating clinical utility of the approach used in this current study. This is also consistent with the finding that KRAS mutation may be a good biomarker of MEK inhibitor sensitivity for treating colorectal cancer [53].

GW-572016 (Lapatinib/Tykerb) is a small-molecule inhibitor of the epidermal growth factor receptor (ErbB1, EGFR) and ErbB2(HER2/neu) tyrosine kinases. It may seem a bit surprising that lapatinib, being an EGFR inhibitor, appears as one of the top hits of GECM results given that the EGFR inhibitors cetuximab and panitumumab are not very effective in treating KRAS-MT advanced CRCs. On the other hand, it is worth noting that the ineffectiveness of those two antibody based EGFR inhibitors does not necessarily mean that other types of EGFR inhibitors, eg. small molecule based ones such as lapatinib, should all be ineffective. This FDA approved drug was developed by GlaxoSmithKline to treat solid tumors like breast and lung cancer. There is a good amount of evidence to suggest that this hit does make a lot of sense. For example, lapatinib plus trametinib in KRAS-MT malignancies is now undergoing a clinical trial (NCT02230553), a phase I/II study with lapatinib plus trametinib in patients with metastatic KRAS-MT colorectal, non-small cell lung and pancreatic cancer [54]. We note here that the two drugs used in this clinical trial, trametinib and lapatinib, happen to be our #1 and #3 top drugs, respectively. Research has found that inhibition of HER2/neu activity may help in treating metastatic colorectal cancer and tumors with mutant KRAS. As lapatinib sensitizes colon cancer cells to EGFR inhibitors or fluoropyrimidines, the combination of lapatinib with standard chemotherapy has been suggested as a new strategy for the treatment of metastatic colorectal cancer [55, 56].

D-cycloserine (cycloserine), the second highest scoring drug, is a novel discovery. Cycloserine is an approved antibiotic drug produced by Streptomyces garyphalus used to treat Mycobacterium avium complex (MAC). Together with up to 5 other drugs, it is also used to treat tuberculosis. There is no evidence in the literature that cycloserine has been investigated in the treatment of colorectal cancer.

In addition to reviewing the drugs individually, it was important to assess whether any drug clusters are overrepresented on the list. This method is similar to pathway or gene set enrichment analysis, but here it is applied to drug groups with similar indications. To our knowledge, this is the first time drug enrichment methods have been employed to interpret and prioritize the predictions from connectivity mapping. Interestingly, we found 7 hypertension drugs among the top 100 drugs, including four antihypertensive angiotensin II receptor blockers (ARBs): eprosartan, irbesartan, losartan and olmesartan within the top 30.

Hypertension (high blood pressure/arterial hypertension) is a chronic medical condition in which the blood pressure in the arteries is persistently elevated. High blood pressure is a known risk factor for cardiovascular disease, the most attributable cause of cardiovascular death [57]. Compared to normal blood pressure, high blood pressure is linked to higher risks of cancer incidence (e.g. oral, colorectal, lung and bladder cancers) in men and cancer death in men and women [58–60]. As antihypertensive medicines, angiotensin-converting-enzyme inhibitors (ACEIs) and antihypertensive angiotensin receptor blockers (ARBs) target the renin-angiotensin system that has also been found to be involved in carcinogenesis by regulating cell proliferation and tumor growth [61]. Renin–angiotensin system inhibitors ACEIs/ARBs might influence tumor angiogenesis by reducing vascular endothelial growth factor expression and induce apoptosis in cancer cells [62]. A systematic review by researchers indicated that ACEIs/ARBs might be associated with a reduced risk of CRC and precancerous lesion. Anti-angiotensin treatments have been found to suppress liver metastasis of colon cancer cells [63]. In addition, work by Makar et al found that long term and high dose use of angiotensin converting enzyme inhibitor (ACEI) and/or angiotensin receptor blockers (ARBs) may be associated with a decreased incidence of colorectal cancer [64]. Furthermore, Engineer et al have found that exposure to angiotensin-converting enzyme inhibitors (ACEIs)/angiotensin receptor blockers (ARBs) and β-adrenoceptor blockers (β-blockers) is associated with improved survival and decreased tumor progression and hospitalizations in patients with advanced colon cancer [65].

For reasons of efficacy and cost, calcium channel blockers and thiazide-type diuretics are chosen as first-line treatments for hypertension, but ACE inhibitors are increasingly being used. For patients under the age of 55 who cannot tolerate ACE inhibitors, an angiotensin II receptor antagonist is recommended as first-line treatment. As antihypertensive medicines, angiotensin-converting-enzyme inhibitors (ACEIs) and antihypertensive angiotensin receptor blockers (ARBs) target the renin-angiotensin system in different ways. ACEIs block enzyme activities of converting the chemical angiotensin I into angiotensin II in the blood. In contrast, ARBs do not prevent the formation of angiotensin II, but instead block receptors of angiotensin II to prevent them from acting on the vessels [66].

Eprosartan, irbesartan, losartan and olmesartan are angiotensin II receptor blockers which appeared in our candidate drug list. As an angiotensin II receptor antagonist, eprosartan blocks the binding of angiotensin II to the AT1 receptor in vascular smooth muscle, among many other tissues. This prompts vasodilation, a reduction in the secretion of vasopressin and aldosterone and consequently leads to the effect of blood pressure decrease. Irbesartan (Aprovel/Karvea/Avapro) may prevent the progression of nephropathy caused by type 2 diabetes, and reduce renal disease development in patients with type 2 diabetes [67]. In cancer studies, irbesartan caused a marked reduction in volume of colorectal cancer liver metastases and caused changes in tumor microvasculature [68]. Losartan may have a positive effect on interrupting progression of diabetic nephropathy and reduction of renal disease progression in patients with type 2 diabetes, hypertension and microalbuminuria or proteinuria [69]. Olmesartan (Benicar/Olmecip/Olsar) is used to treat hypertension individually or in combination with other antihypertensive agents. The side effects of olmesartan for patients of unilateral or bilateral renal artery stenosis include increased serum creatinine or blood urea nitrogen. In 2011, a U.S. Food and Drug Administration safety review concluded that the benefits of Benical (olmesartan) as an antihypertensive agent continue to outweigh its potential risk [70].

Eprosartan, irbesartan, losartan and olmesartan were identified as significant drugs from connectivity mapping with the KRAS mutant query gene signature. These four drugs are known to block Type-1 angiotensin II receptor, encoded by the AGTR1 gene, and hence they are categorized as angiotensin II receptor blockers. As the target of these ARB drugs, AGTR1 plays a major role in controlling blood pressure and volume in the cardiovascular system through its interaction with angiotensin II. AGTR1 itself is not in the combined KRAS-MT gene signature. One possible explanation of the predicted ARB effects on CRCs is that ARB drugs may have other targets in addition to AGTR1, and exert their anti-cancer effects via those unknown targets independently of AGTR1. Alternatively, there may be some yet to be understood molecular mechanism connecting the angiotensin system with cancer development and progression.

To understand why ARB anti-hypertension drugs might be useful for cancer treatment, one notes that the local renin-angiotensin system has been reported to promote angiogenesis and vascular proliferation through the expression of VEGF (vascular endothelial growth factor) or EGFR (epidermal growth factor receptors) [71, 72]. Meanwhile, inducing angiogenesis is a well-known hallmark of cancer [73, 74], as cancer cells need a constant supply of nutrients and oxygen through the blood system to sustain their growth and proliferation. The tumor-associated neovasculature, generated by the process of angiogenesis, addresses these needs of the cancer cells. The facts that the ARB drugs block the angiotensin II receptors and affect the functionalities of the renin-angiotensin system, point to the possibility that ARBs may curb the angiogenesis-promoting activities of the system, and consequently exert their effects on cancer cell growth and proliferation. The detailed molecular mechanism of ARBs on cancer cells is still not well understood, but there seems to be a good amount of evidence for the potential role of the local renin-angiotensin system in carcinogenesis [75], and there are reports on the effect of angiotensin II type-1 receptor (AT1R) antagonists in suppressing the growth of gastric cancer cells [76] and preventing angiogenesis and growth of xenograft tumors developed by human bladder cancer cells [77]. Therefore, the downregulation of AT1R, eg by ARB drugs, may well weaken the angiogenetic and tumor-proliferative effects of angiotensin [78]. These are all consistent with and supportive of our findings.

It is interesting to note that in a recent study [22], Iorio et al developed a semi-supervised iterative pipeline to systematically refine drug-response signatures to identify novel drugs that share the principal mode of action of some given “seed drug”, an approach that was successfully applied to paclitaxel, a microtubule stabilizing agent, as the seed compound. This iterative approach could be used to dissect the disease signatures generated in GECM and may help to tease out the modes of action of the compounds in the GECM output drug list. The integration of this approach with our GECM process is beyond the scope of this current study, but nevertheless, it represents a line of future research which would be interesting to explore.

In this study, we conducted an intensive process to select gene signatures and candidate compounds for KRAS-MT colorectal cancer using a novel approach to GECM. Pathway analyses were used as quality control and biological validation for both selected genes and drugs to ensure the most precise results were obtained after each step. Compounds identified in the connectivity mapping analysis include currently used CRC treatment indicating the power of connectivity mapping and strong connections established between the KRAS-MT signature and the drug list. Angiotensin II receptor antagonists are strong therapeutic candidates emerging from our connectivity mapping analyses, which is interestingly supported by other research in the field.

To the best of our knowledge, this is the first study that has shown the potential of ARB drugs as specific therapies for KRAS-MT CRC. As enrichment methods can provide more objective insights into prioritizing and interpreting the drugs, the result that ARB drugs are highly enriched in the significant drug list lends strong motivational support to further studies investigating ARB drugs as potential therapies for treating KRAS-MT CRCs. For example, to go beyond the scope and limit of the current study, it would be important in future research to investigate these ARB drugs experimentally in KRAS-MT model systems (cell lines and/or animal models) to gain in-depth mechanistic understanding of their actions and interactors, providing a reinforced biological and pharmacological basis for subsequent clinical studies. Such follow-up efforts will undoubtedly facilitate the transition from our computational findings to clinically validated benefits to CRC patients.

## MATERIALS AND METHODS

### Datasets: Sample selection

Datasets GSE35896, GSE39084 and GSE39582 were obtained from the Gene Expression Omnibus (GEO), a global public functional data repository collecting enormous high-throughput functional genomics data (such as microarray and sequencing data) distributed freely to the research community. The selection of these datasets for inclusion in the current study was a result of querying the GEO database using “KRAS” and “colorectal” and either “GPL96” or “GPL570” as keywords, then filtered by GSEs (GEO Data Series) that contain patient clinical samples. GPL96 and GPL570 are two gene expression platforms particularly useful for the gene expression connectivity mapping analysis in this study. GPL96 (HG-U133A, Affymetrix Human Genome U133A Array) is the same microarray platform the Connectivity Map was based on, and GPL570 (HG-U133_Plus_2, Affymetrix Human Genome U133 Plus 2.0 Array) contains almost all the probeIDs present in GPL96. Importantly, all the LINCS gene expression profiles are presented in a set of 22268 probeIDs which are present in both GPL96 and GPL570 platforms. Therefore, using gene expression data from these two platforms, no identifier conversion is needed and little or no information loss is involved in the process. Dataset GSE35896 from Schlicker et al includes 29 KRAS-MT and 33 KRAS-WT totaling 62 samples [79]. GSE39084 was from Kirzin et al's study, in which they collected the information of 954 patients treated and followed-up for CRC at Centre Hospitalier Universitaire de Toulouse, France between April 1999 and December 2005 [80]. Dataset GSE39084 includes on-line available expression data of 70 samples (30 samples are KRAS-MT, 40 samples are KRAS-WT). GSE39582 from the French national Cartes d’Identité des Tumeurs (CIT) program involves 750 CRC patients who underwent surgery between 1987 and 2007 across seven French research centers/hospitals [81]. We used 545 samples with obtainable KRAS information, comprising 217 KRAS-MT and 328 KRAS-WT samples. The platforms used for these datasets were Affymetrix HG-U133 Plus 2 containing the expression measurement of 54675 probes per sample. As an additional note here, we also browsed the TCGA database and found 626 CRC cases, but unfortunately no gene expression data with our selected microarray platforms (Affymetrix HG-U133 plus 2) was available for the CRC cases in TCGA. Nevertheless, TCGA as a valuable data resource should be explored and utilized in future research on other types of cancers where suitable datasets can be found.

### Data processing and analysis

Gene expression raw data of GSE35896, GSE39084 and GSE39582 were downloaded from the GEO website, extracted and read into the R environment (R version 2.15.1). CEL files were then normalized and summarized using the MAS5 algorithm as implemented in the Bioconductor affy package, providing human readable gene expression data (as tabular text files) of the samples. To compare the gene expression of samples between KRAS-MT and KRAS-WT, the GEO series matrix files were downloaded and extracted to obtain the KRAS mutation status of all individual samples. The samples of each dataset were then grouped into a KRAS-MT and a KRAS-WT cluster respectively. We therefore obtained 6 subsets of samples for our analysis. Each dataset now as a unit of analysis consists of two sub-datasets, one for KRAS-MT samples and one for KRAS-WT samples.

### Gene selection

The query gene signature is a panel of genes that needs to be selected to serves as an input to connectivity mapping. These genes exhibit a significant expression change between the conditions of KRAS-MT and KRAS-WT CRCs and are a representation (characteristics) of the biological effects of KRAS mutation on CRC. An unpaired two-sample *t*-test was carried out between the wild type and mutant samples of each dataset, *p*-value and fold-change for each gene were calculated. In order to rigorously control the false discovery rate, a stringent threshold *p*-value (alpha) = 1/n was set, where n is the total number of genes analyzed. As tumor samples are being analyzed and the comparison was carried out between KRAS-MT and KRAS-WT CRCs, the gene expression change of KRAS mutant vs wild type was subtle, so we chose a slightly more inclusive threshold of 1.2 for the fold change filter. A gene that fulfilled these two criteria, namely with a *p*-value smaller than 1/n and a fold change larger than 1.2 (either up or down), was defined as a significant gene. The collection of significant genes from statistical testing of each dataset was primarily sorted in ascending ordering of their *p*-value and secondarily sorted in descending order by the absolute value of log ratio. The gene on the top of the list has the smallest *p*-value, which is the most significant gene with expression change between KRAS-MT and KRAS-WT groups. A sign (+) or (-) is added to each gene according to the direction of gene expression change. As a result, each gene has a sign to indicate if this gene is up-regulated or down-regulated in the KRAS-MT samples, which is crucially important for specifying the gene signature pattern.

To obtain a robust significant genes list and ensure that we have selected genuine gene expression alteration across these different datasets, we bring together significant genes identified from each dataset to create a combined gene signature using a normalized ranking method [82]. Each significant gene has a signed score from each dataset as an indication of its significance and regulation direction, and its signed scores from all datasets were added to give this gene an overall score. If a gene regulates differently in different datasets, the scores with opposite signs will cancel each other to some extent and the total score will be reduced. This method ensures that genes consistently significant across different datasets and also with same regulation direction gain high absolute scores. Genes with a non-zero overall score were selected on the combined signature. The genes in the combined signature were then sorted in descending order by their absolute overall scores, so the most significant gene is on top of the list. In this study, 248 gene probes were included in the combined signature.

### KEGG and Ingenuity pathway analysis

KEGG and Ingenuity Pathway Analysis are additional steps to ensure that the selected genes are relevant to the biological condition we are investigating and to determine the biological relevance of the selected genes. KEGG pathway analysis was carried out using the combined gene signature as initial input. The probe IDs in the gene signature were mapped to Entrez gene IDs using the Affymetrix annotation table. Members of KEGG pathways were also presented as Entrez genes. An over-representation analysis of a pathway in the gene signature was performed for the KEGG human pathways. Briefly, for each KEGG human pathway, the number of probe IDs in the gene signature that can be mapped to this pathway was assessed against a background distribution of all probe IDs used in the differential expression analysis. This background distribution specified the number probe IDs that can be mapped to this particular pathway and the number probe IDs that cannot. All the mappings were conducted via the Entrez gene IDs included in the Affymetrix annotation table. The statistical significance of the number of probe IDs in the gene signature mapped to this pathway was then calculated using a hypergeometric test.

Following differential expression analysis and signature generation, identified probe IDs were also analyzed using QIAGEN's Ingenuity Pathway Analysis (IPA, http://www.ingenuity.com, released in December 2015). Each probe ID was mapped to its gene annotation and the directionality (up or down-regulated) was included for the significantly differentially regulated probes. The returned annotated gene lists were then used to generate a core analysis, resulting in predicted phenotype, upstream regulators and networks alongside functional and canonical pathway analyses. The results from KEGG and Ingenuity Pathway Analysis validated the biological relevance of our selected gene signature to the KRAS-MT CRCs.

### Connectivity mapping procedure

As one of the key components of connectivity mapping, the query gene signature is the input from researchers and the starting point of the connectivity mapping procedure. The quality of gene selection shapes the pattern in the query signature and consequently determines the output drugs. When many genes are identified as significant from the result of statistical hypothesis testing, the strategy used to construct a query signature from this set of genes is critically important. A well-designed query pattern is a vital step in obtaining an accurate profile of the biological condition in question and ultimately getting valuable results from connectivity mapping.

**Figure 6:**
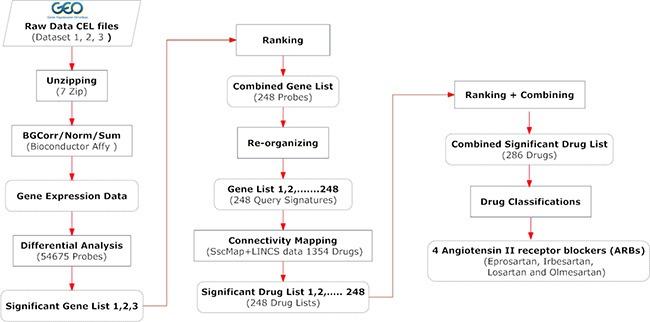
A flowchart of the comprehensive procedure for gene signature creation and drug identification The Materials and Methods section provides more detailed description of the key steps involved.

The common method of executing connectivity mapping is to use one single query signature to represent a biological condition of interest. Researchers normally use the full list of significant genes as the query signature. As gene selection is pivotal for the final drug results, obtaining the best result relies on whether a query signature can describe the true picture of the biological condition. We observed that not only the gene selection but also the length of a query signature influences the drug results. As mentioned above, the normalized ranking method we used helps to select the most relevant genes. In order to obtain precise drug results from connectivity mapping with the selected genes, we propose a novel process for conducting connectivity mapping using a panel of query signatures. Figure 6 shows a flowchart of this procedure.

The prime purpose of this new process is to fully utilize the selected genes while minimizing possible effects of the signature length. The combined KRAS-MT signature of CRC from our statistical analyses on three independent datasets includes 248 significant genes that passed our stringent criteria. Genes on this list were sorted to ensure the most significant gene is on the top. We created 248 derivative signatures: signature 1 contained only the top gene; signature 2 contained the top 2 two genes; signature 3 the top 3 three genes, and so forth, with signature 248 containing the full list of these 248 genes. We carried out connectivity mapping with these 248 query gene signatures individually using the sscMap framework and the reference profiles of 1354 FDA approved drugs from the LINCS database. We consequently obtained 248 drug lists from connectivity mapping as the results for these 248 derived query signatures.

### Drugs selection and prioritization

After connectivity mapping, each individual signature produced a list of significant drugs. The first few signatures, with small numbers of genes, had relatively low numbers of significant drug hits. For instance, query gene signature 1 had only 3 drug hits and signature 2 had 4 drug hits. In order to identify the drugs that are consistently significant across the 248 lists, the significant drugs on each list are sorted in descending order according to the absolute zscore (normalized score) from sscMap. A normalized ranking method was also used to score the significant drugs in each list to ensure that the scores from different lists are comparable. This approach is similar to the ranking method we used for the combined gene signature. Non-significant drugs score 0, while significant drugs’ scores are calculated using the formula below:

Drug score = (M - i + 1)/M

where M is the total number of significant drugs on the list and i is the rank of the drug in the list. The drug score is then signed in line with the original zscore. The signed scores of each drug from all lists were added up in the final combined drug list, where each drug has a sum score of 248 individual scores, one from each drug list. The drugs were then sorted in descending order of the absolute sum score. There were 286 drugs with non-zero sum score in the combined list that may have the potential to treat the disease condition, and the top drug had a negative score of -241.5, the last drug's score was -0.01 (See Supplementary File 7 for the full list of the 286 drugs and their overall scores).

**Figure 7:**
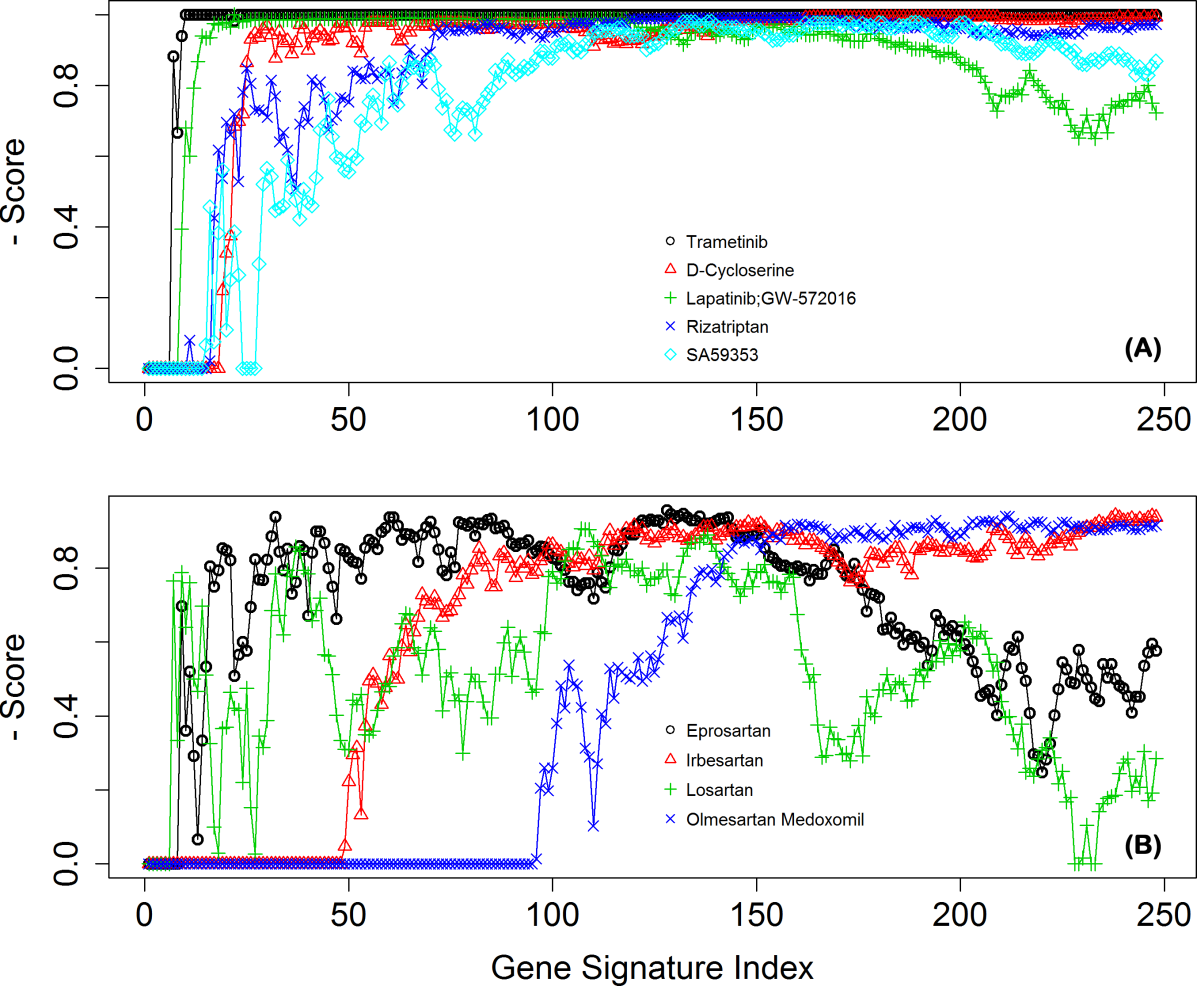
The normalized scores of selected drugs across the 248 derived signatures (**A**) for the top 5 drugs, and (**B**) for the four ARB drugs.

Figure 7A shows the score changes of the identified top 5 drugs across signatures 1 - 248. These drugs consistently gained high scores in the 248 drug lists. In addition to examining top significant drugs, we also looked at recurrent themes among the top 30 candidate compounds by manually examining the occurrence of drugs that have been used to treat the same diseases or that belong to a type of classification, eg, targeting the same proteins or genes in their known mechanism of action. The most prominent drug themes were then selected to undergo quantitative assessment of their statistical significance by a hypergeometric test. The whole collection of 1354 drugs used in the GECM analysis was examined to obtain the number of present drugs with that particular theme (eg, targeting a particular gene). Consequently, the total number 1354 and the number with the drug theme became the background distribution for this particular drug theme. The hypergeometric test was then performed on the occurrence of this drug theme among the top 30 candidate compounds under the described background distribution. From this analysis, we found angiotensin II receptor blockers (ARB drugs) were far more significantly enriched than the other tested themes (EGFR, ERBB2, or MAP2K1/2-targeting drugs) among our top 30 drugs. The score changes of the 4 ARBs drugs across these 248 drug lists are shown in Figure 7B.

### Contributive genes for the antihypertensive drugs

To investigate the reasons that ARB drugs were selected from connectivity mapping, contributive genes analysis was conducted to provide in-depth information of the genes whose regulation was disrupted in response to these drugs. We used the LINCS reference gene expression profiles to investigate which signature genes contributed to the significant connections between the antihypertensive drugs and the KRAS-MT CRC disease state. We analyzed the signed ranks of the signature genes in these reference profiles in conjunction with the corresponding regulation status in the KRAS-MT CRC gene signature. For each drug, the mean signed rank for each probe, across all reference profiles for that drug, was calculated, and then multiplied by the regulation status of the corresponding probe (+1 for up-regulation, −1 for down-regulation) in the gene signature. This allows the individual ‘contribution’ of each probe towards the overall connection score to be found - those probes making a negative contribution are of interest in potentially exposing the mechanism of action of the particular drug under consideration. The four angiotensin receptor blockers (ARB), namely eprosartan, irbesartan, losartan and olmesartan are of particular interest. Venny, which is an interactive tool for comparing lists using Venn Diagrams with support for up to 4 sets, was used to analyze these contributive genes to find the overlaps among the four drugs [34].

### Validation against drug screen sensitivity data

Drug screen sensitivity data were obtained from two independent studies. The inclusion of datasets from these drug screen studies was guided by the availability of raw data as supplied in the Supplementary Information of these papers. In particular, we needed the KRAS mutation status of the cell lines in order to compare mutant versus wild type cell lines. We were successful in obtaining the KRAS mutation status data from the Seashore-Ludlow 2015 [35] and the Garnett 2012 [38] studies, which were subsequently used in this work.

The list of compounds screened in each study was extracted from the supplementary data of these papers. Then they were mapped to the LINCS compounds collection either through the BRD ID (Broad Institute compound ID) in the case of Seashore-Ludlow data, or by compound name or synonyms in the case of Garnett study. For the common set of compounds, their reference gene expression profiles were extracted from the LINCS database, and their sensitivity data were obtained from the corresponding study involved. The IC50 or AUC are the main measures of drug sensitivity in these studies. The smaller the IC50 or AUC values, the more sensitive the cell line was to the drug.

To compare the sensitivity of KRAS MT versus WT CRC cells to a particular compound, the sensitivity data for cells identified as CRC cell lines were first extracted. Based on the KRAS mutation status, the CRC cell lines were then divided into KRAS-MT and KRAS WT groups. The mean IC50 or AUC values were calculated for KRAS MT and KRAS WT cells, respectively, and the sign of their difference indicated whether KRAS MT cells were more sensitive to that particular drug.

## SUPPLEMENTARY MATERIALS AND TABLES










