# Supplementary material for: KRAS mutant colorectal cancer gene signatures identified angiotensin II receptor blockers as potential therapies
Source: Oncotarget. 2016 Dec 10;8(2):3206–25. doi: 10.18632/oncotarget.13884 (PMC5356876; doi:10.18632/oncotarget.13884)
Supplement: Supplementary file 1 [file oncotarget-08-3206-s001.pdf]

KRAS mutant colorectal cancer gene signatures identified angiotensin II receptor blockers as potential therapies

Supplementary Materials

Supplementary Table S1: Date sets used. See Supplementary_Table_S1

Supplementary Table S2: List of 133 unique annotated up–regulated genes in the KRAS–MT gene signature. See Supplementary_Table_S2

Supplementary Table S3: Summary of hypergeometric tests on the enrichment of the target–inhibiting drugs among the top 30 GECM candidates

Drug Target Gene Symbol	AGTR1	EGFR	MAP2K1/2	ERBB2
Drug target name	Type–1 angiotensin II receptor	Epidermal growth factor receptor	Dual specificity mitogen–activated protein kinase kinase 1/2	Receptor tyrosine–protein kinase erbB–2
Hypergeometric test *p* value	6.57E–06	0.00456	0.0438	0.0438
Drug pool size	1354	1354	1354	1354
Number of drugs in the pool inhibiting the target	7	5	2	2
Drug name list 1	candesartan eprosartan irbesartan losartan olmesartan telmisartan valsartan	afatinib gefitinib erlotinib lapatinib lidocaine	trametinib bosutinib	afatinib lapatinib
Number of selected GECM top drugs	30	30	30	30
Number of drugs inhibiting the target among top selected	4	2	1	1
Drug name list 2	eprosartan irbesartan losartan olmesartan	lapatinib lidocaine	trametinib	lapatinib

List 1 gives the names of all drugs inhibiting the particular target in the pool of 1354 FDA drugs; List 2 are drugs in List 1 that made into the top 30 GECM candidate drugs.

Supplementary Table S4: The lists of contributive genes to the four ARB drugs. See Supplementary_ Table_S4

Supplementary Table S5: The top 20 candidate drugs (with absolute mean score > 0.25) predicted by GECM to be effective in KRAS-MT CRCs; As can be seen from the last column Diff, 16 out of these 20 compounds turned out to be more sensitive in KRAS-MT cells than in KRAS-WT cells, as indicated by the AUC values; the smaller the more sensitive

Compound Name	Replicates	Sum Score	Mean Score	Status	TargetGene	KRAS–WT AUC	KRAS–MT AUC	Diff
selumetinib	467	–241.0	–0.972	clinical	MAP2K1;MAP2K2	10.28	8.99	–1.291
trametinib	133	–234.9	–0.947	clinical	MAP2K1;MAP2K2	8.49	6.78	–1.707
WZ4002	308	–217.9	–0.879	probe	EGFR	13.00	13.00	0.001
tanespimycin	194	–207.2	–0.835	clinical	HSP90AA1	10.20	10.29	0.087
lapatinib	244	–205.0	–0.827	FDA	EGFR;ERBB2	12.49	12.75	0.263
neratinib	346	–191.1	–0.770	clinical	EGFR;ERBB2	11.24	10.79	–0.446
cyanoquinoline 11	169	–159.0	–0.641	clinical	MAP3K8	13.50	13.17	–0.329
TG-101348	325	–156.0	–0.629	clinical	JAK2	12.19	12.13	–0.063
saracatinib	304	–149.1	–0.601	clinical	ABL1;SRC	12.88	12.47	–0.404
dexamethasone	238	–146.3	–0.590	FDA	NR3C1	15.30	15.12	–0.177
etoposide	35	–137.1	–0.553	FDA	TOP2A	13.32	13.01	–0.311
dasatinib	243	–119.0	–0.480	FDA	EPHA2;KIT; LCK;SRC;YES1	11.37	10.19	–1.184
cediranib	67	–108.5	–0.437	clinical	FLT1;FLT4;KDR	12.03	11.67	–0.365
canertinib	200	–104.5	–0.421	clinical	EGFR;ERBB2	11.70	11.73	0.022
PF-543	144	–104.1	–0.420	probe	SPHK1	14.27	13.86	–0.406
staurosporine	159	–90.4	–0.365	probe		14.42	14.16	–0.260
epigallocatechin-3-monogallate	127	–76.2	–0.307	probe		13.70	13.49	–0.211
BEC	171	–74.9	–0.302	probe	ARG1;ARG2	15.26	14.45	–0.814
tamatinib	354	–62.2	–0.251	clinical	SYK	14.10	13.73	–0.370
UNC0321	169	–62.1	–0.250	probe	EHMT2	14.95	14.73	–0.226

The accuracy of GECM prediction in the drug's direction of action is 80%.

Supplementary Table S6: Among the common set of 121 drugs between the Garnett and LINCS collections, the top 13 candidate drugs (with absolute mean score > 0.25) predicted by GECM to be effective in KRAS-MT CRCs; As can be seen from the last column Diff, 10 out of these 13 drugs turned out to be more sensitive in KRAS-MT cells than in KRAS-WT cells, as indicated by the IC50 values; the smaller the more sensitive

Drug Name	Replicate	Sum Score	Mean Score	Drug Targets	KRAS-WT-IC50	KRAS-MT-IC50	Diff
PD-0325901	261	–240.2	–0.968	MEK1/2	–1.150	–1.697	–0.548
CI-1040	306	–232.9	–0.939	MEK1/2	2.340	1.683	–0.657
AZD6244	467	–220.4	–0.889	MEK1/2	1.633	1.200	–0.433
Lapatinib	244	–183.8	–0.741	EGFR, ERBB2	4.214	2.991	–1.223
AP-24534	132	–174.7	–0.704	ABL	2.191	1.967	–0.223
AUY922	416	–167.7	–0.676	HSP90	–2.677	–2.637	0.040
PF-562271	231	–144.4	–0.582	FAK	3.014	2.535	–0.479
AZD-0530	304	–139.5	–0.562	SRC, ABL1	4.169	3.108	–1.061
Etoposide	35	–126.9	–0.512	TOP2	0.963	1.656	0.694
Dasatinib	243	–113.7	–0.459	ABL, SRC, KIT, PDGFR	3.877	1.084	–2.793
AZD7762	237	–94.4	–0.381	CHK1/2	–0.072	1.084	1.156
Bosutinib	83	–84.1	–0.339	SRC, ABL, TEC	3.490	3.469	–0.020
BMS-536924	280	–70.5	–0.284	IGF1R	1.366	0.716	–0.651

The accuracy of GECM predictions in the drug's direction of action is 77%

Supplementary Table S7: The list of 286 significant drugs and their overall scores. See Supplementary_Table_S7
