# Supplementary material for: KRAS mutant colorectal cancer gene signatures identified angiotensin II receptor blockers as potential therapies
Source: Oncotarget. 2016 Dec 10;8(2):3206–25. doi: 10.18632/oncotarget.13884 (PMC5356876; doi:10.18632/oncotarget.13884)
Supplement: Supplementary file 2 [file oncotarget-08-3206-s002.docx]

**Date sets used:**

| **Dataset** | **Samples** | **KRAS.MT** | **KRAS.WT** | **Platform** | **Total Probes** |
| --- | --- | --- | --- | --- | --- |
| GSE39084 | 70 | 30 | 40 | Affy HG-U133Plus2 | 54675 |
| GSE39582 | 545 | 217 | 328 | Affy HG-U133Plus2 | 54675 |
| GSE35896 | 62 | 29 | 33 | Affy HG-U133Plus2 | 54675 |

**The full list of significant probes in the KRAS-MT gene signature:**

| **GeneName** | **ProbeID** | **TotalScore** | **Regulation** | **RankOrder** |
| --- | --- | --- | --- | --- |
| LIMA1 | 217892_s_at | 1.4897 | UP | 1 |
| PTPN13 | 204201_s_at | 1.1899 | UP | 2 |
| FOXK2 | 203064_s_at | -1.0000 | DOWN | 3 |
| HOXB6 | 205366_s_at | 1.0000 | UP | 4 |
| UAP1 | 209340_at | 1.0000 | UP | 5 |
| PHLDA1 | 217997_at | 0.9969 | UP | 6 |
| PHLDA1 | 217999_s_at | 0.9939 | UP | 7 |
| RHOBTB3 | 202976_s_at | 0.9908 | UP | 8 |
| PHLDA1 | 217996_at | 0.9878 | UP | 9 |
| DUSP6 | 208891_at | 0.9847 | UP | 10 |
| BMP4 | 211518_s_at | 0.9817 | UP | 11 |
| SERPINB1 | 213572_s_at | 0.9786 | UP | 12 |
| SERPINB1 | 212268_at | 0.9755 | UP | 13 |
| PHLDA1 | 217998_at | 0.9694 | UP | 14 |
| RHOBTB3 | 202975_s_at | 0.9664 | UP | 15 |
| HOXB5 | 205601_s_at | 0.9633 | UP | 16 |
| KRT6A | 209125_at | 0.9602 | UP | 17 |
| TBX3 | 219682_s_at | 0.9572 | UP | 18 |
| TCF7L2 | 216511_s_at | 0.9565 | UP | 19 |
| FHL2 | 202949_s_at | 0.9541 | UP | 20 |
| RHOBTB3 | 216048_s_at | 0.9511 | UP | 21 |
| CA9 | 205199_at | 0.9480 | UP | 22 |
| DUSP4 | 204015_s_at | 0.9450 | UP | 23 |
| KLK11 | 205470_s_at | 0.9419 | UP | 24 |
| CELF2 | 202158_s_at | 0.9388 | UP | 25 |
| TCN1 | 205513_at | 0.9358 | UP | 26 |
| HOXB7 | 216973_s_at | 0.9327 | UP | 27 |
| MARCH3 | 213256_at | 0.9319 | UP | 28 |
| HOXB5 | 205600_x_at | 0.9297 | UP | 29 |
| ASXL1 | 212237_at | -0.9266 | DOWN | 30 |
| PHLDA1 | 218000_s_at | 0.9235 | UP | 31 |
| C11orf95 | 218641_at | -0.9205 | DOWN | 32 |
| TGFBI | 201506_at | 0.9174 | UP | 33 |
| DUSP6 | 208893_s_at | 0.9144 | UP | 34 |
| TCF7L2 | 216035_x_at | 0.9130 | UP | 35 |
| REEP1 | 204365_s_at | -0.9113 | DOWN | 36 |
| HOXB6 | 205365_at | 0.9089 | UP | 37 |
| TFF1 | 205009_at | 0.9083 | UP | 38 |
| OTUB2 | 219369_s_at | 0.9052 | UP | 39 |
| SCPEP1 | 218217_at | -0.9021 | DOWN | 40 |
| MLPH | 218211_s_at | 0.9016 | UP | 41 |
| HOXB7 | 204779_s_at | 0.8991 | UP | 42 |
| C2orf67 | 215046_at | 0.8960 | UP | 43 |
| DUSP6 | 208892_s_at | 0.8930 | UP | 44 |
| CKAP4 | 200999_s_at | 0.8869 | UP | 45 |
| GALNT10 | 212256_at | 0.8838 | UP | 46 |
| KLK10 | 209792_s_at | 0.8807 | UP | 47 |
| SERPINA1 | 202833_s_at | 0.8777 | UP | 48 |
| DUSP4 | 204014_at | 0.8746 | UP | 49 |
| MSX2 | 210319_x_at | 0.8716 | UP | 50 |
| PCDH9 | 219738_s_at | -0.8696 | DOWN | 51 |
| MSX2 | 205555_s_at | 0.8685 | UP | 52 |
| PARVB | 37966_at | -0.8624 | DOWN | 53 |
| SERPINA1 | 211429_s_at | 0.8593 | UP | 54 |
| APOBEC1 | 207158_at | 0.8563 | UP | 55 |
| HOXB9 | 216417_x_at | 0.8532 | UP | 56 |
| ARSJ | 219973_at | 0.8528 | UP | 57 |
| PIPOX | 221605_s_at | -0.8471 | DOWN | 58 |
| IL23A | 220054_at | 0.8440 | UP | 59 |
| ABHD2 | 205566_at | 0.8410 | UP | 60 |
| BACE2 | 217867_x_at | 0.8379 | UP | 61 |
| FTH1 | 214211_at | -0.8349 | DOWN | 62 |
| SLC26A2 | 205097_at | -0.8318 | DOWN | 63 |
| REEP1 | 204364_s_at | -0.8287 | DOWN | 64 |
| C20orf111 | 221954_at | -0.8226 | DOWN | 65 |
| CD44 | 212063_at | 0.8196 | UP | 66 |
| SERPINB5 | 204855_at | 0.8165 | UP | 67 |
| LAMC2 | 202267_at | 0.8135 | UP | 68 |
| CSNK1E | 222015_at | 0.8104 | UP | 69 |
| RGNEF | 219610_at | 0.8073 | UP | 70 |
| MERTK | 206028_s_at | -0.8043 | DOWN | 71 |
| SLC22A4 | 205896_at | -0.8012 | DOWN | 72 |
| ZNHIT1 | 201541_s_at | 0.8000 | UP | 73 |
| CTSL2 | 210074_at | -0.7982 | DOWN | 74 |
| TBXAS1 | 208130_s_at | 0.7951 | UP | 75 |
| SERPINB6 | 211474_s_at | 0.7920 | UP | 76 |
| MAP3K5 | 203836_s_at | 0.7890 | UP | 77 |
| GZMB | 210164_at | -0.7859 | DOWN | 78 |
| GDPD5 | 213343_s_at | -0.7826 | DOWN | 79 |
| PAPSS2 | 203060_s_at | 0.7799 | UP | 80 |
| SCRN1 | 201462_at | -0.7798 | DOWN | 81 |
| TNS3 | 217853_at | -0.7768 | DOWN | 82 |
| C20orf11 | 218448_at | -0.7706 | DOWN | 83 |
| DIDO1 | 218325_s_at | -0.7676 | DOWN | 84 |
| FADS3 | 216080_s_at | -0.7645 | DOWN | 85 |
| ASAP1 | 221039_s_at | -0.7615 | DOWN | 86 |
| BCL2L14 | 221241_s_at | 0.7584 | UP | 87 |
| SPINK4 | 207214_at | 0.7554 | UP | 88 |
| DYRK4 | 212954_at | 0.7523 | UP | 89 |
| CANT1 | 221732_at | 0.7492 | UP | 90 |
| CACNA1C | 211592_s_at | 0.7462 | UP | 91 |
| ARL1 | 201658_at | 0.7391 | UP | 92 |
| C3orf52 | 219474_at | 0.7370 | UP | 93 |
| RBL1 | 205296_at | -0.7339 | DOWN | 94 |
| EGLN3 | 219232_s_at | 0.7268 | UP | 95 |
| POFUT1 | 212349_at | -0.7248 | DOWN | 96 |
| C4BPB | 208209_s_at | 0.7217 | UP | 97 |
| CD44 | 204489_s_at | 0.7187 | UP | 98 |
| TBC1D9 | 212956_at | -0.7156 | DOWN | 99 |
| PTGER2 | 206631_at | 0.7125 | UP | 100 |
| CYP39A1 | 220432_s_at | 0.7095 | UP | 101 |
| C11orf71 | 218789_s_at | -0.7064 | DOWN | 102 |
| KRT6B | 213680_at | 0.7034 | UP | 103 |
| BIN1 | 210201_x_at | -0.6957 | DOWN | 104 |
| LRRC8E | 220174_at | 0.6942 | UP | 105 |
| C20orf111 | 209020_at | -0.6911 | DOWN | 106 |
| ASXL1 | 212234_at | -0.6881 | DOWN | 107 |
| CTSA | 200661_at | -0.6820 | DOWN | 108 |
| VAV2 | 205536_at | -0.6758 | DOWN | 109 |
| LY6E | 202145_at | -0.6728 | DOWN | 110 |
| PLEK2 | 218644_at | 0.6697 | UP | 111 |
| RASAL1 | 219752_at | 0.6667 | UP | 112 |
| TCF12 | 208986_at | 0.6606 | UP | 113 |
| NT5E | 203939_at | 0.6575 | UP | 114 |
| ECM1 | 209365_s_at | 0.6544 | UP | 115 |
| ME1 | 204058_at | 0.6522 | UP | 116 |
| KCNAB2 | 203402_at | -0.6514 | DOWN | 117 |
| TFF3 | 204623_at | 0.6422 | UP | 118 |
| S100A14 | 218677_at | 0.6361 | UP | 119 |
| EPS8L1 | 91826_at | 0.6330 | UP | 120 |
| MMP7 | 204259_at | 0.6300 | UP | 121 |
| L1TD1 | 219955_at | 0.6269 | UP | 122 |
| TH1L | 220607_x_at | -0.6239 | DOWN | 123 |
|  | 216201_at | 0.6208 | UP | 124 |
| DHX35 | 218579_s_at | -0.6177 | DOWN | 125 |
| HOXB3 | 208414_s_at | 0.6147 | UP | 126 |
| TNIK | 213107_at | 0.6116 | UP | 127 |
| MAP3K5 | 203837_at | 0.6086 | UP | 128 |
| PARVB | 37965_at | -0.6055 | DOWN | 129 |
| CPD | 201943_s_at | -0.6000 | DOWN | 130 |
| FAM169A | 213954_at | 0.5902 | UP | 131 |
| ABHD2 | 87100_at | 0.5872 | UP | 132 |
| NAAA | 214765_s_at | -0.5780 | DOWN | 133 |
| HYAL1 | 210619_s_at | 0.5749 | UP | 134 |
| ANXA2P2 | 208816_x_at | 0.5719 | UP | 135 |
| TRIM16 | 204341_at | 0.5657 | UP | 136 |
| KIAA1199 | 212942_s_at | 0.5627 | UP | 137 |
| HOXB8 | 221278_at | 0.5621 | UP | 138 |
| KLK6 | 204733_at | 0.5566 | UP | 139 |
| C8orf4 | 218541_s_at | 0.5535 | UP | 140 |
| LYZ | 213975_s_at | 0.5505 | UP | 141 |
| ENPP1 | 205066_s_at | -0.5413 | DOWN | 142 |
| EFNB2 | 202668_at | 0.5382 | UP | 143 |
| CYP3A5 | 214234_s_at | 0.5352 | UP | 144 |
| CYP3A5 | 205765_at | 0.5321 | UP | 145 |
| CA8 | 220234_at | 0.5260 | UP | 146 |
| HOXB7 | 204778_x_at | 0.5229 | UP | 147 |
| TCF7L2 | 216037_x_at | 0.5217 | UP | 148 |
| KRT6B | 209126_x_at | 0.5199 | UP | 149 |
| CELF2 | 202157_s_at | 0.5107 | UP | 150 |
| ACN9 | 218981_at | 0.5076 | UP | 151 |
| WASF3 | 204042_at | -0.5015 | DOWN | 152 |
| KLK8 | 206125_s_at | 0.4985 | UP | 153 |
| PITX1 | 209587_at | 0.4954 | UP | 154 |
| ACSF2 | 218844_at | -0.4924 | DOWN | 155 |
| MERTK | 211913_s_at | -0.4893 | DOWN | 156 |
| CTSE | 205927_s_at | 0.4862 | UP | 157 |
| PPP1R3D | 204554_at | -0.4832 | DOWN | 158 |
| NPEPL1 | 218822_s_at | -0.4801 | DOWN | 159 |
| SLC35A1 | 203306_s_at | 0.4783 | UP | 160 |
| SIRPA | 202896_s_at | -0.4740 | DOWN | 161 |
| FABP3 | 214285_at | -0.4679 | DOWN | 162 |
| DSN1 | 219512_at | -0.4618 | DOWN | 163 |
| CKAP4 | 200998_s_at | 0.4526 | UP | 164 |
| SIRPA | 202897_at | -0.4495 | DOWN | 165 |
| KRT6A /// KRT6B /// KRT6C | 214580_x_at | 0.4404 | UP | 166 |
| CREB3L1 | 213059_at | 0.4348 | UP | 167 |
| PLAGL2 | 202925_s_at | -0.4281 | DOWN | 168 |
| TTC9 | 213172_at | 0.4128 | UP | 169 |
| PITX1 | 208502_s_at | 0.4098 | UP | 170 |
| PARVB | 204629_at | -0.4037 | DOWN | 171 |
| ZNF813 | 217665_at | -0.3976 | DOWN | 172 |
| HGD | 205221_at | 0.3914 | UP | 173 |
| GDPD5 | 32502_at | -0.3913 | DOWN | 174 |
| EHHADH | 205222_at | 0.3884 | UP | 175 |
| KCNN4 | 204401_at | 0.3639 | UP | 176 |
| NEU1 | 208926_at | -0.3609 | DOWN | 177 |
| CPS1 | 204920_at | 0.3578 | UP | 178 |
| COL17A1 | 204636_at | 0.3486 | UP | 179 |
| ABHD2 | 63825_at | 0.3456 | UP | 180 |
| NPDC1 | 218086_at | 0.3425 | UP | 181 |
| DSG3 | 205595_at | 0.3303 | UP | 182 |
| BTG3 | 215425_at | -0.3272 | DOWN | 183 |
| HDGFRP3 | 209524_at | -0.3211 | DOWN | 184 |
| NINL | 207705_s_at | -0.3180 | DOWN | 185 |
| KLK10 | 215808_at | 0.3150 | UP | 186 |
| ALDH3A1 | 205623_at | 0.3119 | UP | 187 |
| FABP3 | 205738_s_at | -0.3089 | DOWN | 188 |
| IL33 | 209821_at | 0.3058 | UP | 189 |
| ABCC3 | 214979_at | 0.3043 | UP | 190 |
| SLC26A3 | 215657_at | -0.2997 | DOWN | 191 |
| CBLC | 220638_s_at | 0.2936 | UP | 192 |
| RBMS1 | 215127_s_at | -0.2905 | DOWN | 193 |
| KNG1 | 206054_at | -0.2844 | DOWN | 194 |
| C20orf46 | 219958_at | -0.2752 | DOWN | 195 |
| NUP210 | 212316_at | -0.2722 | DOWN | 196 |
| ST6GAL1 | 201998_at | -0.2661 | DOWN | 197 |
| DKK4 | 206619_at | 0.2630 | UP | 198 |
| BIN1 | 202931_x_at | -0.2609 | DOWN | 199 |
| ABHD2 | 221815_at | 0.2538 | UP | 200 |
| NMNAT2 | 209755_at | -0.2508 | DOWN | 201 |
| SORBS1 | 218087_s_at | -0.2477 | DOWN | 202 |
| LPAR6 | 218589_at | 0.2355 | UP | 203 |
| PLA2G3 | 220780_at | 0.2232 | UP | 204 |
| NEDD9 | 202149_at | 0.2171 | UP | 205 |
| MAP2K6 | 205698_s_at | 0.2141 | UP | 206 |
| SLC6A14 | 219795_at | 0.2110 | UP | 207 |
| CRYM | 205489_at | 0.2080 | UP | 208 |
| GNAS | 214157_at | -0.2018 | DOWN | 209 |
| CLTB | 206284_x_at | 0.2000 | UP | 210 |
| SULT2B1 | 205759_s_at | 0.1927 | UP | 211 |
| E2F1 | 204947_at | -0.1896 | DOWN | 212 |
| IL1A | 210118_s_at | 0.1804 | UP | 213 |
| DMD | 203881_s_at | -0.1774 | DOWN | 214 |
| PAPSS2 | 203058_s_at | 0.1743 | UP | 215 |
| KLK7 | 205778_at | 0.1468 | UP | 216 |
| C11orf9 | 204073_s_at | 0.1437 | UP | 217 |
| FCGBP | 203240_at | 0.1376 | UP | 218 |
| FADS3 | 204257_at | -0.1346 | DOWN | 219 |
| MTHFD2L | 220346_at | -0.1315 | DOWN | 220 |
| RHOD | 209885_at | -0.1284 | DOWN | 221 |
| ERN2 | 214372_x_at | 0.1254 | UP | 222 |
| FAIM2 | 203619_s_at | 0.1223 | UP | 223 |
| SELE | 206211_at | -0.1131 | DOWN | 224 |
| ZBTB10 | 219312_s_at | -0.1070 | DOWN | 225 |
| CADM1 | 209031_at | -0.1040 | DOWN | 226 |
| MB | 204179_at | 0.1009 | UP | 227 |
| KRT7 | 209016_s_at | 0.0887 | UP | 228 |
| KCNK1 | 204678_s_at | 0.0870 | UP | 229 |
| TBC1D9 | 212960_at | -0.0856 | DOWN | 230 |
| EPB41L2 | 201718_s_at | 0.0826 | UP | 231 |
| MIA3 | 212310_at | 0.0765 | UP | 232 |
| GMDS | 214106_s_at | 0.0734 | UP | 233 |
| DKK1 | 204602_at | 0.0642 | UP | 234 |
| ITGB6 | 208083_s_at | 0.0612 | UP | 235 |
| MUC2 | 204673_at | 0.0581 | UP | 236 |
| SERPINE2 | 212190_at | 0.0550 | UP | 237 |
| EPHB3 | 1438_at | 0.0489 | UP | 238 |
| SLC14A1 | 205856_at | 0.0459 | UP | 239 |
| HGD | 214307_at | 0.0398 | UP | 240 |
| TOX | 204529_s_at | 0.0367 | UP | 241 |
| FLRT3 | 219250_s_at | 0.0336 | UP | 242 |
| CES1 | 209616_s_at | -0.0306 | DOWN | 243 |
| EPS8L1 | 221665_s_at | 0.0183 | UP | 244 |
| RAB7L1 | 218700_s_at | -0.0122 | DOWN | 245 |
| CDC42EP2 | 214014_at | 0.0092 | UP | 246 |
| CD55 | 201925_s_at | 0.0061 | UP | 247 |
| FADS1 | 208962_s_at | -0.0031 | DOWN | 248 |
