# Supplementary material for: KRAS mutant colorectal cancer gene signatures identified angiotensin II receptor blockers as potential therapies
Source: Oncotarget. 2016 Dec 10;8(2):3206–25. doi: 10.18632/oncotarget.13884 (PMC5356876; doi:10.18632/oncotarget.13884)
Supplement: Supplementary file 3 [file oncotarget-08-3206-s003.docx]

**List of 133 unique annotated up-regulated genes in the KRAS-MT gene signature**

| **Symbol** | **MatchedProbes** | **MatchedCount** | **Entrez Gene Name** | **Location** | **Type(s)** |
| --- | --- | --- | --- | --- | --- |
| PHLDA1 | 217996_at, 217997_at, 217998_at, 217999_s_at, 218000_s_at | 5 | pleckstrin homology-like domain, family A, member 1 | Cytoplasm | other |
| ABHD2 | 205566_at, 221815_at, 63825_at, 87100_at | 4 | abhydrolase domain containing 2 | Other | enzyme |
| DUSP6 | 208891_at, 208892_s_at, 208893_s_at | 3 | dual specificity phosphatase 6 | Cytoplasm | phosphatase |
| HOXB7 | 204778_x_at, 204779_s_at, 216973_s_at | 3 | homeobox B7 | Nucleus | transcription regulator |
| RHOBTB3 | 202975_s_at, 202976_s_at, 216048_s_at | 3 | Rho-related BTB domain containing 3 | Cytoplasm | enzyme |
| TCF7L2 | 216035_x_at, 216037_x_at, 216511_s_at | 3 | transcription factor 7-like 2 (T-cell specific, HMG-box) | Nucleus | transcription regulator |
| CD44 | 204489_s_at, 212063_at | 2 | CD44 molecule (Indian blood group) | Plasma Membrane | enzyme |
| CELF2 | 202157_s_at, 202158_s_at | 2 | CUGBP, Elav-like family member 2 | Nucleus | other |
| CKAP4 | 200998_s_at, 200999_s_at | 2 | cytoskeleton-associated protein 4 | Cytoplasm | other |
| CYP3A5 | 205765_at, 214234_s_at | 2 | cytochrome P450, family 3, subfamily A, polypeptide 5 | Cytoplasm | enzyme |
| DUSP4 | 204014_at, 204015_s_at | 2 | dual specificity phosphatase 4 | Nucleus | phosphatase |
| EPS8L1 | 221665_s_at, 91826_at | 2 | EPS8-like 1 | Cytoplasm | other |
| HGD | 205221_at, 214307_at | 2 | homogentisate 1,2-dioxygenase | Cytoplasm | enzyme |
| HOXB5 | 205600_x_at, 205601_s_at | 2 | homeobox B5 | Nucleus | transcription regulator |
| HOXB6 | 205365_at, 205366_s_at | 2 | homeobox B6 | Nucleus | transcription regulator |
| KLK10 | 209792_s_at, 215808_at | 2 | kallikrein-related peptidase 10 | Extracellular Space | peptidase |
| KRT6B | 209126_x_at, 213680_at | 2 | keratin 6B, type II | Cytoplasm | other |
| MAP3K5 | 203836_s_at, 203837_at | 2 | mitogen-activated protein kinase kinase kinase 5 | Cytoplasm | kinase |
| MSX2 | 205555_s_at, 210319_x_at | 2 | msh homeobox 2 | Nucleus | transcription regulator |
| PAPSS2 | 203058_s_at, 203060_s_at | 2 | 3'-phosphoadenosine 5'-phosphosulfate synthase 2 | Cytoplasm | enzyme |
| PITX1 | 208502_s_at, 209587_at | 2 | paired-like homeodomain 1 | Nucleus | transcription regulator |
| SERPINA1 | 202833_s_at, 211429_s_at | 2 | serpin peptidase inhibitor, clade A (alpha-1 antiproteinase, antitrypsin), member 1 | Extracellular Space | other |
| SERPINB1 | 212268_at, 213572_s_at | 2 | serpin peptidase inhibitor, clade B (ovalbumin), member 1 | Cytoplasm | other |
| MARCH3 | 213256_at | 1 | membrane-associated ring finger (C3HC4) 3, E3 ubiquitin protein ligase | Cytoplasm | other |
| ALDH3A1 | 205623_at | 1 | aldehyde dehydrogenase 3 family, member A1 | Cytoplasm | enzyme |
| ANXA2P2 | 208816_x_at | 1 | annexin A2 pseudogene 2 | Cytoplasm | other |
| APOBEC1 | 207158_at | 1 | apolipoprotein B mRNA editing enzyme, catalytic polypeptide 1 | Cytoplasm | enzyme |
| ARHGEF28 | 219610_at | 1 | Rho guanine nucleotide exchange factor (GEF) 28 | Cytoplasm | other |
| ARL1 | 201658_at | 1 | ADP-ribosylation factor-like 1 | Cytoplasm | enzyme |
| ARSJ | 219973_at | 1 | arylsulfatase family, member J | Extracellular Space | enzyme |
| BACE2 | 217867_x_at | 1 | beta-site APP-cleaving enzyme 2 | Cytoplasm | peptidase |
| BCL2L14 | 221241_s_at | 1 | BCL2-like 14 (apoptosis facilitator) | Cytoplasm | other |
| BMP4 | 211518_s_at | 1 | bone morphogenetic protein 4 | Extracellular Space | growth factor |
| C3orf52 | 219474_at | 1 | chromosome 3 open reading frame 52 | Other | other |
| C4BPB | 208209_s_at | 1 | complement component 4 binding protein, beta | Extracellular Space | other |
| C8orf4 | 218541_s_at | 1 | chromosome 8 open reading frame 4 | Nucleus | other |
| CA8 | 220234_at | 1 | carbonic anhydrase VIII | Cytoplasm | enzyme |
| CA9 | 205199_at | 1 | carbonic anhydrase IX | Nucleus | enzyme |
| CACNA1C | 211592_s_at | 1 | calcium channel, voltage-dependent, L type, alpha 1C subunit | Plasma Membrane | ion channel |
| CANT1 | 221732_at | 1 | calcium activated nucleotidase 1 | Extracellular Space | enzyme |
| CBLC | 220638_s_at | 1 | Cbl proto-oncogene C, E3 ubiquitin protein ligase | Nucleus | enzyme |
| CD55 | 201925_s_at | 1 | CD55 molecule, decay accelerating factor for complement (Cromer blood group) | Plasma Membrane | other |
| CDC42EP2 | 214014_at | 1 | CDC42 effector protein (Rho GTPase binding) 2 | Plasma Membrane | other |
| CEMIP | 212942_s_at | 1 | cell migration inducing protein, hyaluronan binding | Cytoplasm | enzyme |
| CLTB | 206284_x_at | 1 | clathrin, light chain B | Plasma Membrane | other |
| COL17A1 | 204636_at | 1 | collagen, type XVII, alpha 1 | Extracellular Space | other |
| CPS1 | 204920_at | 1 | carbamoyl-phosphate synthase 1, mitochondrial | Cytoplasm | enzyme |
| CREB3L1 | 213059_at | 1 | cAMP responsive element binding protein 3-like 1 | Nucleus | transcription regulator |
| CRYM | 205489_at | 1 | crystallin, mu | Cytoplasm | enzyme |
| CSNK1E | 222015_at | 1 | casein kinase 1, epsilon | Cytoplasm | kinase |
| CTSE | 205927_s_at | 1 | cathepsin E | Cytoplasm | peptidase |
| CYP39A1 | 220432_s_at | 1 | cytochrome P450, family 39, subfamily A, polypeptide 1 | Cytoplasm | enzyme |
| DKK1 | 204602_at | 1 | dickkopf WNT signaling pathway inhibitor 1 | Extracellular Space | growth factor |
| DKK4 | 206619_at | 1 | dickkopf WNT signaling pathway inhibitor 4 | Extracellular Space | other |
| DSG3 | 205595_at | 1 | desmoglein 3 | Plasma Membrane | other |
| DYRK4 | 212954_at | 1 | dual-specificity tyrosine-(Y)-phosphorylation regulated kinase 4 | Nucleus | kinase |
| ECM1 | 209365_s_at | 1 | extracellular matrix protein 1 | Extracellular Space | transporter |
| EFNB2 | 202668_at | 1 | ephrin-B2 | Plasma Membrane | other |
| EGLN3 | 219232_s_at | 1 | egl-9 family hypoxia-inducible factor 3 | Cytoplasm | enzyme |
| EHHADH | 205222_at | 1 | enoyl-CoA, hydratase/3-hydroxyacyl CoA dehydrogenase | Cytoplasm | enzyme |
| EPB41L2 | 201718_s_at | 1 | erythrocyte membrane protein band 4.1-like 2 | Plasma Membrane | other |
| EPHB3 | 1438_at | 1 | EPH receptor B3 | Plasma Membrane | kinase |
| ERN2 | 214372_x_at | 1 | endoplasmic reticulum to nucleus signaling 2 | Cytoplasm | kinase |
| FAIM2 | 203619_s_at | 1 | Fas apoptotic inhibitory molecule 2 | Plasma Membrane | other |
| FAM169A | 213954_at | 1 | family with sequence similarity 169, member A | Other | other |
| FCGBP | 203240_at | 1 | Fc fragment of IgG binding protein | Extracellular Space | other |
| FHL2 | 202949_s_at | 1 | four and a half LIM domains 2 | Nucleus | transcription regulator |
| FLRT3 | 219250_s_at | 1 | fibronectin leucine rich transmembrane protein 3 | Plasma Membrane | other |
| GALNT10 | 212256_at | 1 | polypeptide N-acetylgalactosaminyltransferase 10 | Cytoplasm | enzyme |
| GMDS | 214106_s_at | 1 | GDP-mannose 4,6-dehydratase | Cytoplasm | enzyme |
| HOXB3 | 208414_s_at | 1 | homeobox B3 | Nucleus | transcription regulator |
| HOXB8 | 221278_at | 1 | homeobox B8 | Nucleus | transcription regulator |
| HOXB9 | 216417_x_at | 1 | homeobox B9 | Nucleus | transcription regulator |
| HYAL1 | 210619_s_at | 1 | hyaluronoglucosaminidase 1 | Cytoplasm | enzyme |
| IL1A | 210118_s_at | 1 | interleukin 1, alpha | Extracellular Space | cytokine |
| IL23A | 220054_at | 1 | interleukin 23, alpha subunit p19 | Extracellular Space | cytokine |
| IL33 | 209821_at | 1 | interleukin 33 | Extracellular Space | cytokine |
| ITGB6 | 208083_s_at | 1 | integrin, beta 6 | Plasma Membrane | other |
| KANSL1L | 215046_at | 1 | KAT8 regulatory NSL complex subunit 1-like | Other | other |
| KCNK1 | 204678_s_at | 1 | potassium channel, two pore domain subfamily K, member 1 | Plasma Membrane | ion channel |
| KCNN4 | 204401_at | 1 | potassium channel, calcium activated intermediate/small conductance subfamily N alpha, member 4 | Plasma Membrane | ion channel |
| KLK11 | 205470_s_at | 1 | kallikrein-related peptidase 11 | Extracellular Space | peptidase |
| KLK6 | 204733_at | 1 | kallikrein-related peptidase 6 | Extracellular Space | peptidase |
| KLK7 | 205778_at | 1 | kallikrein-related peptidase 7 | Extracellular Space | peptidase |
| KLK8 | 206125_s_at | 1 | kallikrein-related peptidase 8 | Extracellular Space | peptidase |
| KRT6A | 209125_at | 1 | keratin 6A, type II | Cytoplasm | other |
| KRT7 | 209016_s_at | 1 | keratin 7, type II | Cytoplasm | other |
| L1TD1 | 219955_at | 1 | LINE-1 type transposase domain containing 1 | Other | other |
| LAMC2 | 202267_at | 1 | laminin, gamma 2 | Extracellular Space | other |
| LIMA1 | 217892_s_at | 1 | LIM domain and actin binding 1 | Cytoplasm | other |
| LPAR6 | 218589_at | 1 | lysophosphatidic acid receptor 6 | Plasma Membrane | G-protein coupled receptor |
| LRRC8E | 220174_at | 1 | leucine rich repeat containing 8 family, member E | Other | other |
| LYZ | 213975_s_at | 1 | lysozyme | Extracellular Space | enzyme |
| MAP2K6 | 205698_s_at | 1 | mitogen-activated protein kinase kinase 6 | Cytoplasm | kinase |
| MB | 204179_at | 1 | myoglobin | Cytoplasm | transporter |
| ME1 | 204058_at | 1 | malic enzyme 1, NADP(+)-dependent, cytosolic | Cytoplasm | enzyme |
| MIA3 | 212310_at | 1 | melanoma inhibitory activity family, member 3 | Cytoplasm | other |
| MLPH | 218211_s_at | 1 | melanophilin | Cytoplasm | other |
| MMP7 | 204259_at | 1 | matrix metallopeptidase 7 | Extracellular Space | peptidase |
| MUC2 | 204673_at | 1 | mucin 2, oligomeric mucus/gel-forming | Other | other |
| MYRF | 204073_s_at | 1 | myelin regulatory factor | Nucleus | transcription regulator |
| NEDD9 | 202149_at | 1 | neural precursor cell expressed, developmentally down-regulated 9 | Nucleus | other |
| NPDC1 | 218086_at | 1 | neural proliferation, differentiation and control, 1 | Extracellular Space | other |
| NT5E | 203939_at | 1 | 5'-nucleotidase, ecto (CD73) | Plasma Membrane | phosphatase |
| OTUB2 | 219369_s_at | 1 | OTU deubiquitinase, ubiquitin aldehyde binding 2 | Nucleus | enzyme |
| PLA2G3 | 220780_at | 1 | phospholipase A2, group III | Extracellular Space | enzyme |
| PLEK2 | 218644_at | 1 | pleckstrin 2 | Other | other |
| PTGER2 | 206631_at | 1 | prostaglandin E receptor 2 (subtype EP2), 53kDa | Plasma Membrane | G-protein coupled receptor |
| PTPN13 | 204201_s_at | 1 | protein tyrosine phosphatase, non-receptor type 13 (APO-1/CD95 (Fas)-associated phosphatase) | Cytoplasm | phosphatase |
| RASAL1 | 219752_at | 1 | RAS protein activator like 1 (GAP1 like) | Cytoplasm | other |
| S100A14 | 218677_at | 1 | S100 calcium binding protein A14 | Cytoplasm | other |
| SDHAF3 | 218981_at | 1 | succinate dehydrogenase complex assembly factor 3 | Cytoplasm | other |
| SERPINB5 | 204855_at | 1 | serpin peptidase inhibitor, clade B (ovalbumin), member 5 | Extracellular Space | other |
| SERPINB6 | 211474_s_at | 1 | serpin peptidase inhibitor, clade B (ovalbumin), member 6 | Cytoplasm | other |
| SERPINE2 | 212190_at | 1 | serpin peptidase inhibitor, clade E (nexin, plasminogen activator inhibitor type 1), member 2 | Extracellular Space | other |
| SLC14A1 | 205856_at | 1 | solute carrier family 14 (urea transporter), member 1 (Kidd blood group) | Plasma Membrane | transporter |
| SLC35A1 | 203306_s_at | 1 | solute carrier family 35 (CMP-sialic acid transporter), member A1 | Cytoplasm | transporter |
| SLC6A14 | 219795_at | 1 | solute carrier family 6 (amino acid transporter), member 14 | Plasma Membrane | transporter |
| SPINK4 | 207214_at | 1 | serine peptidase inhibitor, Kazal type 4 | Extracellular Space | other |
| SULT2B1 | 205759_s_at | 1 | sulfotransferase family, cytosolic, 2B, member 1 | Cytoplasm | enzyme |
| TBX3 | 219682_s_at | 1 | T-box 3 | Nucleus | transcription regulator |
| TBXAS1 | 208130_s_at | 1 | thromboxane A synthase 1 (platelet) | Plasma Membrane | enzyme |
| TCF12 | 208986_at | 1 | transcription factor 12 | Nucleus | transcription regulator |
| TCN1 | 205513_at | 1 | transcobalamin I (vitamin B12 binding protein, R binder family) | Cytoplasm | transporter |
| TFF1 | 205009_at | 1 | trefoil factor 1 | Extracellular Space | other |
| TFF3 | 204623_at | 1 | trefoil factor 3 (intestinal) | Extracellular Space | other |
| TGFBI | 201506_at | 1 | transforming growth factor, beta-induced, 68kDa | Extracellular Space | other |
| TNIK | 213107_at | 1 | TRAF2 and NCK interacting kinase | Plasma Membrane | kinase |
| TOX | 204529_s_at | 1 | thymocyte selection-associated high mobility group box | Nucleus | other |
| TRIM16 | 204341_at | 1 | tripartite motif containing 16 | Cytoplasm | transcription regulator |
| TTC9 | 213172_at | 1 | tetratricopeptide repeat domain 9 | Extracellular Space | other |
| UAP1 | 209340_at | 1 | UDP-N-acetylglucosamine pyrophosphorylase 1 | Nucleus | enzyme |
| ZNHIT1 | 201541_s_at | 1 | zinc finger, HIT-type containing 1 | Nucleus | other |

**List of 68 unique annotated down-regulated genes in the KRAS-MT gene signature**

| **Symbol** | **MatchedProbes** | **MatchedCount** | **Entrez Gene Name** | **Location** | **Type(s)** |
| --- | --- | --- | --- | --- | --- |
| PARVB | 204629_at, 37965_at, 37966_at | 3 | parvin, beta | Cytoplasm | other |
| ASXL1 | 212234_at, 212237_at | 2 | additional sex combs like transcriptional regulator 1 | Nucleus | transcription regulator |
| BIN1 | 202931_x_at, 210201_x_at | 2 | bridging integrator 1 | Nucleus | other |
| FABP3 | 205738_s_at, 214285_at | 2 | fatty acid binding protein 3, muscle and heart | Cytoplasm | transporter |
| FADS3 | 204257_at, 216080_s_at | 2 | fatty acid desaturase 3 | Plasma Membrane | enzyme |
| GDPD5 | 213343_s_at, 32502_at | 2 | glycerophosphodiester phosphodiesterase domain containing 5 | Plasma Membrane | enzyme |
| MERTK | 206028_s_at, 211913_s_at | 2 | MER proto-oncogene, tyrosine kinase | Plasma Membrane | kinase |
| OSER1 | 209020_at, 221954_at | 2 | oxidative stress responsive serine-rich 1 | Other | enzyme |
| REEP1 | 204364_s_at, 204365_s_at | 2 | receptor accessory protein 1 | Cytoplasm | other |
| SIRPA | 202896_s_at, 202897_at | 2 | signal-regulatory protein alpha | Plasma Membrane | phosphatase |
| TBC1D9 | 212956_at, 212960_at | 2 | TBC1 domain family, member 9 (with GRAM domain) | Plasma Membrane | other |
| ACSF2 | 218844_at | 1 | acyl-CoA synthetase family member 2 | Cytoplasm | enzyme |
| ASAP1 | 221039_s_at | 1 | ArfGAP with SH3 domain, ankyrin repeat and PH domain 1 | Plasma Membrane | other |
| BTG3 | 215425_at | 1 | BTG family, member 3 | Nucleus | other |
| C11orf71 | 218789_s_at | 1 | chromosome 11 open reading frame 71 | Other | other |
| C11orf95 | 218641_at | 1 | chromosome 11 open reading frame 95 | Other | other |
| CADM1 | 209031_at | 1 | cell adhesion molecule 1 | Plasma Membrane | other |
| CES1 | 209616_s_at | 1 | carboxylesterase 1 | Cytoplasm | enzyme |
| CPD | 201943_s_at | 1 | carboxypeptidase D | Extracellular Space | peptidase |
| CTSA | 200661_at | 1 | cathepsin A | Cytoplasm | peptidase |
| CTSV | 210074_at | 1 | cathepsin V | Cytoplasm | peptidase |
| DHX35 | 218579_s_at | 1 | DEAH (Asp-Glu-Ala-His) box polypeptide 35 | Other | enzyme |
| DIDO1 | 218325_s_at | 1 | death inducer-obliterator 1 | Nucleus | other |
| DMD | 203881_s_at | 1 | dystrophin | Plasma Membrane | other |
| DSN1 | 219512_at | 1 | DSN1 homolog, MIS12 kinetochore complex component | Nucleus | other |
| E2F1 | 204947_at | 1 | E2F transcription factor 1 | Nucleus | transcription regulator |
| ENPP1 | 205066_s_at | 1 | ectonucleotide pyrophosphatase/phosphodiesterase 1 | Plasma Membrane | enzyme |
| FADS1 | 208962_s_at | 1 | fatty acid desaturase 1 | Plasma Membrane | enzyme |
| FOXK2 | 203064_s_at | 1 | forkhead box K2 | Nucleus | transcription regulator |
| FTH1 | 214211_at | 1 | ferritin, heavy polypeptide 1 | Cytoplasm | enzyme |
| GID8 | 218448_at | 1 | GID complex subunit 8 | Nucleus | other |
| GNAS | 214157_at | 1 | GNAS complex locus | Plasma Membrane | enzyme |
| GZMB | 210164_at | 1 | granzyme B (granzyme 2, cytotoxic T-lymphocyte-associated serine esterase 1) | Cytoplasm | peptidase |
| HDGFRP3 | 209524_at | 1 | hepatoma-derived growth factor, related protein 3 | Nucleus | other |
| KCNAB2 | 203402_at | 1 | potassium channel, voltage gated subfamily A regulatory beta subunit 2 | Plasma Membrane | ion channel |
| KNG1 | 206054_at | 1 | kininogen 1 | Extracellular Space | other |
| LY6E | 202145_at | 1 | lymphocyte antigen 6 complex, locus E | Plasma Membrane | other |
| MTHFD2L | 220346_at | 1 | methylenetetrahydrofolate dehydrogenase (NADP+ dependent) 2-like | Cytoplasm | enzyme |
| NAAA | 214765_s_at | 1 | N-acylethanolamine acid amidase | Cytoplasm | enzyme |
| NELFCD | 220607_x_at | 1 | negative elongation factor complex member C/D | Nucleus | other |
| NEU1 | 208926_at | 1 | sialidase 1 (lysosomal sialidase) | Cytoplasm | enzyme |
| NINL | 207705_s_at | 1 | ninein-like | Cytoplasm | other |
| NMNAT2 | 209755_at | 1 | nicotinamide nucleotide adenylyltransferase 2 | Cytoplasm | enzyme |
| NPEPL1 | 218822_s_at | 1 | aminopeptidase-like 1 | Nucleus | peptidase |
| NUP210 | 212316_at | 1 | nucleoporin 210kDa | Nucleus | transporter |
| PCDH9 | 219738_s_at | 1 | protocadherin 9 | Plasma Membrane | other |
| PIPOX | 221605_s_at | 1 | pipecolic acid oxidase | Cytoplasm | enzyme |
| PLAGL2 | 202925_s_at | 1 | pleiomorphic adenoma gene-like 2 | Nucleus | transcription regulator |
| POFUT1 | 212349_at | 1 | protein O-fucosyltransferase 1 | Cytoplasm | enzyme |
| PPP1R3D | 204554_at | 1 | protein phosphatase 1, regulatory subunit 3D | Cytoplasm | phosphatase |
| RAB29 | 218700_s_at | 1 | RAB29, member RAS oncogene family | Plasma Membrane | enzyme |
| RBL1 | 205296_at | 1 | retinoblastoma-like 1 | Nucleus | transcription regulator |
| RBMS1 | 215127_s_at | 1 | RNA binding motif, single stranded interacting protein 1 | Nucleus | other |
| RHOD | 209885_at | 1 | ras homolog family member D | Cytoplasm | enzyme |
| SCPEP1 | 218217_at | 1 | serine carboxypeptidase 1 | Cytoplasm | peptidase |
| SCRN1 | 201462_at | 1 | secernin 1 | Cytoplasm | other |
| SELE | 206211_at | 1 | selectin E | Plasma Membrane | transmembrane receptor |
| SLC22A4 | 205896_at | 1 | solute carrier family 22 (organic cation/zwitterion transporter), member 4 | Plasma Membrane | transporter |
| SLC26A2 | 205097_at | 1 | solute carrier family 26 (anion exchanger), member 2 | Plasma Membrane | transporter |
| SLC26A3 | 215657_at | 1 | solute carrier family 26 (anion exchanger), member 3 | Plasma Membrane | transporter |
| SORBS1 | 218087_s_at | 1 | sorbin and SH3 domain containing 1 | Plasma Membrane | other |
| ST6GAL1 | 201998_at | 1 | ST6 beta-galactosamide alpha-2,6-sialyltranferase 1 | Cytoplasm | enzyme |
| TMEM74B | 219958_at | 1 | transmembrane protein 74B | Other | other |
| TNS3 | 217853_at | 1 | tensin 3 | Plasma Membrane | phosphatase |
| VAV2 | 205536_at | 1 | vav 2 guanine nucleotide exchange factor | Cytoplasm | transcription regulator |
| WASF3 | 204042_at | 1 | WAS protein family, member 3 | Cytoplasm | other |
| ZBTB10 | 219312_s_at | 1 | zinc finger and BTB domain containing 10 | Nucleus | other |
| ZNF813 | 217665_at | 1 | zinc finger protein 813 | Other | Other |

**Upstream regulators of the genes in the KRAS-MT gene signatures**

| **Upstream Regulator** | **Molecule Type** | **Predicted State** | **Activation z-score** | **p-value of overlap** | **Target molecules in data set** |
| --- | --- | --- | --- | --- | --- |
| TGFB1 | growth factor | Activated | 2.565 | 3.83E-08 | BMP4, CD44, CD55, CELF2, CEMIP, DUSP4, ECM1, HOXB5, IL1A, IL23A, IL33, ITGB6, KLK11, KRT7, LAMC2, MIA3, MMP7, NEDD9, NT5E, PLEK2, PTGER2, SERPINA1, SERPINB1, SERPINB5, SERPINE2, SLC35A1, TBX3, TCF12, TGFBI |
| EGF | growth factor | Activated | 2.035 | 0.00712 | CD44, DUSP4, DUSP6, HOXB5, MB, MUC2, SERPINA1, TFF1 |
| HGF | growth factor | Activated | 2.761 | 0.00756 | CA9, CD44, DUSP4, DUSP6, EFNB2, FAIM2, LAMC2, PHLDA1 |
| IGF1 | growth factor | Activated | 2.318 | 0.0278 | BMP4, CD44, EFNB2, PHLDA1, TFF1, TGFBI |
