# Supplementary material for: KRAS mutant colorectal cancer gene signatures identified angiotensin II receptor blockers as potential therapies
Source: Oncotarget. 2016 Dec 10;8(2):3206–25. doi: 10.18632/oncotarget.13884 (PMC5356876; doi:10.18632/oncotarget.13884)
Supplement: Supplementary file 4 [file oncotarget-08-3206-s004.docx]

**The lists of contributive genes to the four ARB drugs**

| **eprosartan** | **irbesartan** | **losartan** | **olmesartan** |
| --- | --- | --- | --- |
| ABHD2 | APOBEC1 | CD44 | MMP7 |
| ALDH3A1 | KRT6B | BCL2L14 | RHOBTB3 |
| APOBEC1 | DUSP6 | SERPINB5 | PLEK2 |
| ARSJ | C3orf52 | MB | ZNHIT1 |
| ASXL1 | CD44 | PTGER2 | CBLC |
| BACE2 | CD55 | ERN2 | MUC2 |
| BCL2L14 | HOXB6 | BACE2 | GDPD5 |
| BMP4 | ANXA2P2 | ECM1 | HOXB3 |
| BTG3 | EFNB2 | ACSF2 | EHHADH |
| C11orf95 | EPB41L2 | PAPSS2 | ABHD2 |
| C20orf111 | CADM1 | PARVB | MAP3K5 |
| C20orf46 | MAP3K5 | CTSE | ARSJ |
| C3orf52 | MTHFD2L | MAP3K5 | SLC35A1 |
| C8orf4 | IL23A | C20orf111 | IL33 |
| CD44 | L1TD1 | FTH1 | SERPINA1 |
| CD55 | RHOD | PTPN13 | REEP1 |
| CDC42EP2 | REEP1 | BMP4 | HOXB7 |
| CES1 | TFF3 | GDPD5 | HOXB5 |
| CKAP4 | LAMC2 | HOXB7 | TBX3 |
| CLTB | NUP210 | FOXK2 | KLK10 |
| CPS1 | PHLDA1 | ARL1 | CA9 |
| CREB3L1 | C20orf111 | MTHFD2L | FABP3 |
| CRYM | KLK10 | ME1 | ZBTB10 |
| CTSA | ST6GAL1 | CES1 | WASF3 |
| CTSE | CYP3A5 | C3orf52 | ABCC3 |
| CYP39A1 | ENPP1 | SLC26A2 | DSG3 |
| DHX35 | RGNEF | DKK1 | CRYM |
| DIDO1 | LRRC8E | FHL2 | BTG3 |
| DKK4 | PAPSS2 | FADS1 | TFF3 |
| DMD | SLC26A2 | EHHADH | KNG1 |
| DSG3 | MERTK | EPB41L2 | DHX35 |
| DUSP4 | BTG3 | PPP1R3D | RGNEF |
| DUSP6 | TTC9 | GMDS | GZMB |
| E2F1 | SLC35A1 | TCF7L2 | HGD |
| ECM1 | DKK1 | APOBEC1 | TBC1D9 |
| EFNB2 | PLA2G3 | NEDD9 | PIPOX |
| ENPP1 | MARCH3 | CELF2 | KIAA1199 |
| EPS8L1 | CPS1 | MARCH3 | KRT6B |
| ERN2 | OTUB2 | C2orf67 | NMNAT2 |
| FADS3 | PARVB | ITGB6 | TOX |
| FAIM2 | COL17A1 | WASF3 | C20orf11 |
| FHL2 | KIAA1199 | NMNAT2 | KLK11 |
| GDPD5 | KCNAB2 | C20orf46 | NAAA |
| GNAS | KRT7 | CKAP4 | SLC26A2 |
| HGD | TNS3 | BIN1 | PTGER2 |
| HOXB3 | ACSF2 | TRIM16 | HOXB6 |
| HOXB5 | RASAL1 | CACNA1C | E2F1 |
| HOXB6 | SERPINB6 | ZBTB10 | OTUB2 |
| HOXB7 | EPHB3 | L1TD1 | TNS3 |
| HOXB9 | ABHD2 | GNAS | BMP4 |
| IL1A | RBMS1 | LPAR6 | CYP3A5 |
| IL33 | CDC42EP2 | ASXL1 | BIN1 |
| ITGB6 | GMDS | TNIK | DSN1 |
| KCNK1 | BCL2L14 | HGD | HYAL1 |
| KIAA1199 | GALNT10 | RGNEF | C11orf71 |
| KLK11 | TNIK | DYRK4 | C3orf52 |
| KLK6 | CREB3L1 | ACN9 | KLK6 |
| KNG1 | SORBS1 | MUC2 | CTSE |
| KRT6A /// KRT6B /// KRT6C | TBX3 | LYZ | C4BPB |
| KRT6B | FHL2 | RHOBTB3 | SULT2B1 |
| KRT7 | SERPINB1 | RAB7L1 | FADS1 |
| L1TD1 | KLK6 | FAM169A | DUSP4 |
| LAMC2 | PITX1 | RBMS1 | PITX1 |
| LIMA1 | NT5E | HOXB9 | RASAL1 |
| LRRC8E | DKK4 | ARSJ | RAB7L1 |
| LYZ | CTSL2 | KNG1 | MARCH3 |
| MAP3K5 | CSNK1E | ASAP1 | MSX2 |
| MB | C4BPB | HOXB8 | LYZ |
| ME1 | IL33 | FADS3 | TRIM16 |
| MIA3 | VAV2 | HOXB6 | C2orf67 |
| MLPH | TRIM16 | HYAL1 | S100A14 |
| MMP7 | ME1 | DUSP4 | MIA3 |
| MSX2 | MLPH | IL23A | SPINK4 |
| MUC2 | PLEK2 | PHLDA1 | CLTB |
| NAAA | RBL1 | CSNK1E | TCF12 |
| NINL | ZNHIT1 | LIMA1 | LIMA1 |
| NPEPL1 | HOXB3 | C11orf95 | SELE |
| NT5E | DSG3 | TBXAS1 | ME1 |
| OTUB2 | ZBTB10 | ABHD2 | EPHB3 |
| PHLDA1 | HOXB9 | PITX1 | TNIK |
| PIPOX | CPD | TFF1 | KLK8 |
| PLAGL2 | TBC1D9 | FABP3 | CKAP4 |
| PLEK2 | CA8 | EPS8L1 | PCDH9 |
| POFUT1 | KLK7 | ST6GAL1 | RBL1 |
| PTGER2 | CTSE | RHOD | HOXB9 |
| PTPN13 | C11orf9 | TBC1D9 | CADM1 |
| RAB7L1 | SELE | POFUT1 | SERPINE2 |
| RASAL1 | FAIM2 | KRT6B | CACNA1C |
| RBL1 | DSN1 | CD55 | NUP210 |
| RBMS1 | MAP2K6 | SPINK4 | RBMS1 |
| REEP1 | TCN1 | CBLC | KLK7 |
| RGNEF | LYZ | NAAA | TCF7L2 |
| RHOBTB3 | FLRT3 | FCGBP | SCRN1 |
| RHOD | MSX2 | ABCC3 | KRT7 |
| S100A14 | DUSP4 | KIAA1199 | APOBEC1 |
| SCRN1 | SERPINA1 | CREB3L1 | EPS8L1 |
| SELE | CBLC | IL33 | CD44 |
| SERPINA1 | C2orf67 | TFF3 | KCNAB2 |
| SERPINB1 | SLC6A14 | PLAGL2 | ALDH3A1 |
| SERPINE2 | SERPINE2 | C4BPB | ASAP1 |
| SLC26A3 | TBXAS1 | SCRN1 | BCL2L14 |
| SLC35A1 | NAAA | HOXB5 | C11orf9 |
| SLC6A14 | DYRK4 | CTSA | CD55 |
| SORBS1 | TGFBI | NINL | CELF2 |
| ST6GAL1 | MUC2 | CPD | COL17A1 |
| SULT2B1 | HOXB5 | HOXB3 | CPS1 |
| TBX3 | FOXK2 | CTSL2 | CYP39A1 |
| TBXAS1 | SCRN1 | ANXA2P2 | DUSP6 |
| TCN1 | HDGFRP3 | SLC14A1 | DYRK4 |
| TFF1 | GZMB | SCPEP1 | EFNB2 |
| TFF3 | HGD | REEP1 | EGLN3 |
| TH1L | CKAP4 | CA9 | ENPP1 |
| TNS3 | NEU1 | COL17A1 | GMDS |
| TRIM16 | EPS8L1 | DSG3 | HDGFRP3 |
| TTC9 | SLC26A3 | UAP1 | KRT6A /// KRT6B /// KRT6C |
| VAV2 | SERPINB5 | NPEPL1 | LPAR6 |
| ZBTB10 | NMNAT2 | EPHB3 | MERTK |
|  | ABCC3 | CYP3A5 | PARVB |
|  | CELF2 |  | TH1L |
|  | CRYM | PCDH9 | SERPINB5 |
|  | CYP39A1 |  | SORBS1 |
|  | EHHADH | C11orf71 | NEU1 |
|  | FTH1 |  | SLC6A14 |
|  | HOXB7 | HDGFRP3 | NT5E |
|  | MIA3 | PIPOX | NEDD9 |
|  | NEDD9 | MSX2 | POFUT1 |
|  | SPINK4 | NEU1 | NPEPL1 |
|  | TCF12 |  | TFF1 |
|  | TFF1 |  | SERPINB6 |
|  |  |  | TTC9 |
