# Supplementary material for: KRAS mutant colorectal cancer gene signatures identified angiotensin II receptor blockers as potential therapies
Source: Oncotarget. 2016 Dec 10;8(2):3206–25. doi: 10.18632/oncotarget.13884 (PMC5356876; doi:10.18632/oncotarget.13884)
Supplement: Supplementary file 5 [file oncotarget-08-3206-s005.docx]

**The list of 286 significant drugs and their overall scores.**

| **Drug** | **SumScore** | **absSumScore** | **MeanScore** |
| --- | --- | --- | --- |
| trametinib, JTP-74057;GSK-1120212 | -241.5 | 241.5 | -0.97 |
| d-cycloserine | -220.3 | 220.3 | -0.89 |
| GW-572016, Lapatinib,Tykerb | -220.0 | 220.0 | -0.89 |
| rizatriptan | -213.2 | 213.2 | -0.86 |
| SA59353 | -192.9 | 192.9 | -0.78 |
| selegiline | -192.5 | 192.5 | -0.78 |
| bosentan | -191.2 | 191.2 | -0.77 |
| tolterodine | -188.9 | 188.9 | -0.76 |
| irinotecan hcl )trihydrate) | -187.0 | 187.0 | -0.75 |
| budesonide | -179.9 | 179.9 | -0.73 |
| ponatinib;AP24534 | -178.8 | 178.8 | -0.72 |
| eprosartan | -177.1 | 177.1 | -0.71 |
| donepezil | -175.5 | 175.5 | -0.71 |
| tetrahydrobiopterin | -172.8 | 172.8 | -0.70 |
| metaproterenol | -171.9 | 171.9 | -0.69 |
| irbesartan | -163.0 | 163.0 | -0.66 |
| brimonidine | -157.3 | 157.3 | -0.63 |
| caffeine | -146.5 | 146.5 | -0.59 |
| meropenem | -145.2 | 145.2 | -0.59 |
| lidocaine | -133.3 | 133.3 | -0.54 |
| granisetron | -131.1 | 131.1 | -0.53 |
| losartan | -129.0 | 129.0 | -0.52 |
| nitrazepam | -124.5 | 124.5 | -0.50 |
| phentermine | -123.8 | 123.8 | -0.50 |
| cefazolin | -123.2 | 123.2 | -0.50 |
| 3,5-diamino-N-carbamimidoyl-6-chloropyrazine-2-carboxamide KAJ-25 | -122.5 | 122.5 | -0.49 |
| midazolam hcl | -120.0 | 120.0 | -0.48 |
| olmesartan medoxomil | -119.9 | 119.9 | -0.48 |
| SA792815 | -119.6 | 119.6 | -0.48 |
| erythromycin | -118.7 | 118.7 | -0.48 |
| moban | -117.1 | 117.1 | -0.47 |
| fluocinolone acetonide | -115.3 | 115.3 | -0.46 |
| norpace | -114.8 | 114.8 | -0.46 |
| benazepril hcl | -114.2 | 114.2 | -0.46 |
| terbinafine | -114.2 | 114.2 | -0.46 |
| clocortolone pivalate | -113.1 | 113.1 | -0.46 |
| gefitinib;ZD1839 | -112.7 | 112.7 | -0.45 |
| dofetilide | -108.8 | 108.8 | -0.44 |
| dichlorphenamide | -107.9 | 107.9 | -0.43 |
| tubocurarine | -107.8 | 107.8 | -0.43 |
| zolpidem | -107.4 | 107.4 | -0.43 |
| pralidoxime chloride | -106.6 | 106.6 | -0.43 |
| CPT 11 | -100.4 | 100.4 | -0.40 |
| benztropine mesylate | -100.3 | 100.3 | -0.40 |
| sorafenib tosylate | -100.0 | 100.0 | -0.40 |
| venlafaxine hcl | -99.6 | 99.6 | -0.40 |
| SA1444140 | -99.3 | 99.3 | -0.40 |
| fluticasone | -97.3 | 97.3 | -0.39 |
| azacitidine | -94.9 | 94.9 | -0.38 |
| amlexanox | -94.1 | 94.1 | -0.38 |
| beclometasone | -91.9 | 91.9 | -0.37 |
| fexofenadine | -91.2 | 91.2 | -0.37 |
| adapalene | -90.3 | 90.3 | -0.36 |
| liothyronine | -89.3 | 89.3 | -0.36 |
| diphenoxylate | -87.8 | 87.8 | -0.35 |
| diloxanide | -86.9 | 86.9 | -0.35 |
| flucloxacillin sodium | -86.3 | 86.3 | -0.35 |
| dilantin | -86.2 | 86.2 | -0.35 |
| famciclovir | -85.9 | 85.9 | -0.35 |
| demeclocycline | -85.8 | 85.8 | -0.35 |
| S1014 | -84.0 | 84.0 | -0.34 |
| betazole hydrochloride | -83.7 | 83.7 | -0.34 |
| beclomethasone | -83.5 | 83.5 | -0.34 |
| fenoldopam | -82.6 | 82.6 | -0.33 |
| SA792148 | -82.4 | 82.4 | -0.33 |
| cilastatin na | -82.0 | 82.0 | -0.33 |
| etoposide | -81.9 | 81.9 | -0.33 |
| amcinonide | -81.7 | 81.7 | -0.33 |
| valaciclovir | -81.1 | 81.1 | -0.33 |
| pancuronium | -78.2 | 78.2 | -0.32 |
| pioglitazone hydrochloride | -76.6 | 76.6 | -0.31 |
| sulpiride | -76.2 | 76.2 | -0.31 |
| oxybutynin | -74.8 | 74.8 | -0.30 |
| pramipexole hcl | -73.9 | 73.9 | -0.30 |
| procarbazine | -69.8 | 69.8 | -0.28 |
| desoximetasone | -68.9 | 68.9 | -0.28 |
| troleandomycin | -68.6 | 68.6 | -0.28 |
| rosiglitazone hydrochloride | -66.1 | 66.1 | -0.27 |
| cyclobenzaprine hydrochloride | -63.6 | 63.6 | -0.26 |
| nizatidine | -62.7 | 62.7 | -0.25 |
| propantheline bromide | -62.3 | 62.3 | -0.25 |
| pravastatin | -62.0 | 62.0 | -0.25 |
| milrinone | -61.3 | 61.3 | -0.25 |
| labetalol | -60.7 | 60.7 | -0.24 |
| (+-)-2-(3,4-Dihydroxybenzyl)-2-hydrazinopropionic acid | -60.2 | 60.2 | -0.24 |
| glyburide | -59.0 | 59.0 | -0.24 |
| dasatinib, BMS-354825, sprycel, BMS354825 | -58.6 | 58.6 | -0.24 |
| sibutramine | -57.5 | 57.5 | -0.23 |
| metaraminol bitartrate | -57.0 | 57.0 | -0.23 |
| griseofulvin | -57.0 | 57.0 | -0.23 |
| SA36479 | -49.6 | 49.6 | -0.20 |
| roxatidine hcl | -49.5 | 49.5 | -0.20 |
| etoricoxib | -46.0 | 46.0 | -0.19 |
| cephalexin | -42.5 | 42.5 | -0.17 |
| ticlopidine hcl | -42.3 | 42.3 | -0.17 |
| atorvastatin | -42.0 | 42.0 | -0.17 |
| tinidazole | -41.9 | 41.9 | -0.17 |
| guaifenesin | -41.7 | 41.7 | -0.17 |
| urecholine | -41.5 | 41.5 | -0.17 |
| sotalol | -40.9 | 40.9 | -0.17 |
| betaxolol | -40.4 | 40.4 | -0.16 |
| ganciclovir | -40.1 | 40.1 | -0.16 |
| testosterone | -38.3 | 38.3 | -0.15 |
| atovaquone | -38.2 | 38.2 | -0.15 |
| naloxone | -36.7 | 36.7 | -0.15 |
| novobiocin sodium salt | -35.7 | 35.7 | -0.14 |
| mecamylamine hydrochloride | -33.6 | 33.6 | -0.14 |
| viomycin sulfate | -33.2 | 33.2 | -0.13 |
| (+-)-Fluoxetine hydrochloride | -33.1 | 33.1 | -0.13 |
| adenine 9-beta;-d-arabinofuranoside | -32.9 | 32.9 | -0.13 |
| methimazole | -32.9 | 32.9 | -0.13 |
| acepromazine | -32.8 | 32.8 | -0.13 |
| terazosin | -32.3 | 32.3 | -0.13 |
| tolmetin | -32.0 | 32.0 | -0.13 |
| midodrine hydrochloride | -30.3 | 30.3 | -0.12 |
| penciclovir | -28.7 | 28.7 | -0.12 |
| sulindac | -28.7 | 28.7 | -0.12 |
| fluticasone propionate | -28.4 | 28.4 | -0.11 |
| (+-)-(N,N-Dimethyl)ephedrine Iodide | -28.1 | 28.1 | -0.11 |
| SA59772 | -26.2 | 26.2 | -0.11 |
| pyrazinamide | -26.1 | 26.1 | -0.11 |
| aminolevulinic acid | -26.0 | 26.0 | -0.11 |
| ritonavir | -26.0 | 26.0 | -0.10 |
| epirubicin | -25.5 | 25.5 | -0.10 |
| quinethazone | -25.4 | 25.4 | -0.10 |
| chlorambucil | -25.2 | 25.2 | -0.10 |
| rifaximin | -24.8 | 24.8 | -0.10 |
| dirithromycin | -23.5 | 23.5 | -0.095 |
| chloroquine diphosphate | -23.3 | 23.3 | -0.094 |
| erlotinib;osi-774 | -23.2 | 23.2 | -0.093 |
| rivastigmine | -23.1 | 23.1 | -0.093 |
| quinidine | -22.5 | 22.5 | -0.091 |
| dabrafenib | -22.1 | 22.1 | -0.089 |
| remoxipride | -20.5 | 20.5 | -0.083 |
| mesoridazine | -20.2 | 20.2 | -0.082 |
| cortisone | -20.2 | 20.2 | -0.081 |
| flumazenil | -19.6 | 19.6 | -0.079 |
| isotretinoin | -19.1 | 19.1 | -0.077 |
| clarithromycin | -18.7 | 18.7 | -0.075 |
| loteprednol | -18.1 | 18.1 | -0.073 |
| terbinafine hcl | -17.4 | 17.4 | -0.070 |
| moxifloxacin hcl | -16.1 | 16.1 | -0.065 |
| 1,3,5(10)-estratrien-3-ol-17-one sulphate | -16.1 | 16.1 | -0.065 |
| buspar | -15.6 | 15.6 | -0.063 |
| oxaprozin | -15.3 | 15.3 | -0.062 |
| donepezil hydrochloride | -14.9 | 14.9 | -0.060 |
| clindamycin hydrochloride | -14.9 | 14.9 | -0.060 |
| metoclopramide | -14.6 | 14.6 | -0.059 |
| Lenalidomide | -14.5 | 14.5 | -0.059 |
| alverine | -14.5 | 14.5 | -0.058 |
| s(-)-timolol | -14.3 | 14.3 | -0.058 |
| meclomen | -13.2 | 13.2 | -0.053 |
| irinotecan | -13.1 | 13.1 | -0.053 |
| medrysone | -13.1 | 13.1 | -0.053 |
| nefazodone | -12.6 | 12.6 | -0.051 |
| amoxicillin crystalline | -12.5 | 12.5 | -0.050 |
| ceforanide | -12.0 | 12.0 | -0.049 |
| indomethacin | -11.9 | 11.9 | -0.048 |
| Moricizine hydrochloride | -11.6 | 11.6 | -0.047 |
| famotidine | -11.1 | 11.1 | -0.045 |
| ketorolac tromethamine | -9.27 | 9.27 | -0.037 |
| Lovastatin | -9.18 | 9.18 | -0.037 |
| bendroflumethiazide | -9.00 | 9.00 | -0.036 |
| rifapentine | -8.70 | 8.70 | -0.035 |
| fexofenadine hydrochloride | -8.59 | 8.59 | -0.035 |
| cefaclor | -7.86 | 7.86 | -0.032 |
| loratadine | -7.52 | 7.52 | -0.030 |
| idarubicin hcl | -7.51 | 7.51 | -0.030 |
| rosiglitazone hcl | -7.30 | 7.30 | -0.029 |
| clotrimazole | -7.29 | 7.29 | -0.029 |
| hyoscyamine | -7.26 | 7.26 | -0.029 |
| SAHA | -7.10 | 7.10 | -0.029 |
| estriol | -6.91 | 6.91 | -0.028 |
| tamoxifen citrate | -6.86 | 6.86 | -0.028 |
| ranitidine | -6.62 | 6.62 | -0.027 |
| nevirapine | -6.46 | 6.46 | -0.026 |
| dextromethorphan hydrobromide | -6.42 | 6.42 | -0.026 |
| finasteride | -6.35 | 6.35 | -0.026 |
| felbamate | -6.08 | 6.08 | -0.025 |
| mometasone furoate | -5.64 | 5.64 | -0.023 |
| phenylbutazone | -5.62 | 5.62 | -0.023 |
| hexylcaine | -5.48 | 5.48 | -0.022 |
| zonisamide | -5.31 | 5.31 | -0.021 |
| dactinomycin | -5.09 | 5.09 | -0.021 |
| propylthiouracil | -4.82 | 4.82 | -0.019 |
| clemastine fumarate | -4.78 | 4.78 | -0.019 |
| dantrolene | -4.67 | 4.67 | -0.019 |
| warfarin | -4.32 | 4.32 | -0.017 |
| pramipexole | -4.21 | 4.21 | -0.017 |
| solifenacin succinate | -4.15 | 4.15 | -0.017 |
| zalcitabine | -4.02 | 4.02 | -0.016 |
| tiabendazole | -3.99 | 3.99 | -0.016 |
| irinotecan hydrochloride trihydrate | -3.85 | 3.85 | -0.016 |
| flurandrenolide | -3.69 | 3.69 | -0.015 |
| metolazone | -3.67 | 3.67 | -0.015 |
| topotecan hcl | -3.60 | 3.60 | -0.015 |
| molindone hydrochloride | -3.35 | 3.35 | -0.014 |
| dibenzyline | -3.15 | 3.15 | -0.013 |
| ethosuximide | -3.14 | 3.14 | -0.013 |
| viramune | -3.12 | 3.12 | -0.013 |
| ropivacaine hcl | -3.11 | 3.11 | -0.013 |
| acarbose | -3.11 | 3.11 | -0.013 |
| pidorubicine | -3.09 | 3.09 | -0.012 |
| loperamide.hcl | -2.99 | 2.99 | -0.012 |
| rosuvastatin calcium | -2.85 | 2.85 | -0.011 |
| tolazamide | -2.83 | 2.83 | -0.011 |
| cefoxitin | -2.77 | 2.77 | -0.011 |
| bisoprolol | -2.63 | 2.63 | -0.011 |
| lymecycline | -2.62 | 2.62 | -0.011 |
| moexipril hydrochloride | -2.28 | 2.28 | -9.2E-03 |
| phenelzine | -2.24 | 2.24 | -9.0E-03 |
| reserpine | -2.21 | 2.21 | -8.9E-03 |
| flurbiprofen | -2.18 | 2.18 | -8.8E-03 |
| pilocarpine nitrate | -2.07 | 2.07 | -8.4E-03 |
| S1189 | -2.00 | 2.00 | -8.1E-03 |
| timolol maleate salt | -1.94 | 1.94 | -7.8E-03 |
| S1025 | -1.63 | 1.63 | -6.6E-03 |
| S1005 | -1.61 | 1.61 | -6.5E-03 |
| methoxsalen | -1.57 | 1.57 | -6.3E-03 |
| betamethasone | -1.54 | 1.54 | -6.2E-03 |
| altretamine | -1.51 | 1.51 | -6.1E-03 |
| balsalazide | -1.49 | 1.49 | -6.0E-03 |
| loperamide | -1.42 | 1.42 | -5.7E-03 |
| theophylline monohydrate | -1.38 | 1.38 | -5.6E-03 |
| fluvastatin | -1.36 | 1.36 | -5.5E-03 |
| hy-10181 | -1.34 | 1.34 | -5.4E-03 |
| aminophylline | -1.28 | 1.28 | -5.1E-03 |
| piroxicam | -1.25 | 1.25 | -5.1E-03 |
| gefitinib | -1.20 | 1.20 | -4.9E-03 |
| S1011 | -1.12 | 1.12 | -4.5E-03 |
| ramipril | -1.03 | 1.03 | -4.2E-03 |
| prednisolone | -1.03 | 1.03 | -4.1E-03 |
| cefpodoxime proxetil | -1.02 | 1.02 | -4.1E-03 |
| rimexolone | -1.00 | 1.00 | -4.0E-03 |
| alclometasone | -0.91 | 0.91 | -3.7E-03 |
| meclofenamic-acid | -0.90 | 0.90 | -3.6E-03 |
| amfebutamone hcl | -0.88 | 0.88 | -3.5E-03 |
| carbimazole | -0.84 | 0.84 | -3.4E-03 |
| pentoxifylline | -0.75 | 0.75 | -3.0E-03 |
| norethisterone | -0.67 | 0.67 | -2.7E-03 |
| trifluridine | -0.66 | 0.66 | -2.7E-03 |
| evista | -0.66 | 0.66 | -2.7E-03 |
| cetirizine dihydrochloride | -0.65 | 0.65 | -2.6E-03 |
| 4-aminosalicylic acid | -0.65 | 0.65 | -2.6E-03 |
| levocabastine hydrochloride | -0.57 | 0.57 | -2.3E-03 |
| equilin | -0.57 | 0.57 | -2.3E-03 |
| miglitol | -0.56 | 0.56 | -2.3E-03 |
| flunisolide | -0.56 | 0.56 | -2.3E-03 |
| pseudoephedrine | -0.51 | 0.51 | -2.1E-03 |
| melatonin | -0.50 | 0.50 | -2.0E-03 |
| clonidine.hcl | -0.50 | 0.50 | -2.0E-03 |
| cilostazol | -0.50 | 0.50 | -2.0E-03 |
| domperidone | -0.42 | 0.42 | -1.7E-03 |
| hydrocortisone | -0.42 | 0.42 | -1.7E-03 |
| oxacillin sodium | -0.42 | 0.42 | -1.7E-03 |
| cytarabine | -0.40 | 0.40 | -1.6E-03 |
| tranexamic acid | -0.36 | 0.36 | -1.5E-03 |
| itavastatin ca | -0.34 | 0.34 | -1.4E-03 |
| terbutaline | -0.28 | 0.28 | -1.1E-03 |
| dicycloverine | -0.27 | 0.27 | -1.1E-03 |
| lincomycin hydrochloride | -0.26 | 0.26 | -1.0E-03 |
| 5-fluorouracil;51-21-8 | -0.25 | 0.25 | -1.0E-03 |
| alprazolam | -0.24 | 0.24 | -9.6E-04 |
| vorinostat | -0.23 | 0.23 | -9.1E-04 |
| prednicarbate | -0.22 | 0.22 | -8.7E-04 |
| EPA | -0.18 | 0.18 | -7.4E-04 |
| imatinib mesylate | -0.18 | 0.18 | -7.3E-04 |
| pioglitazone hcl | -0.18 | 0.18 | -7.2E-04 |
| fluorometholone | -0.13 | 0.13 | -5.4E-04 |
| duloxetine hcl | -0.13 | 0.13 | -5.4E-04 |
| auranofin | -0.13 | 0.13 | -5.3E-04 |
| diazoxide | -0.12 | 0.12 | -4.9E-04 |
| toremiphene citrate | -0.11 | 0.11 | -4.4E-04 |
| thalidomide | -0.10 | 0.10 | -3.9E-04 |
| acamprosate | -0.09 | 0.09 | -3.7E-04 |
| metformin.hcl | -0.06 | 0.06 | -2.6E-04 |
| trioxsalen | -0.06 | 0.06 | -2.4E-04 |
| ritodrine hydrochloride | -0.06 | 0.06 | -2.4E-04 |
| fluvoxamine | -0.05 | 0.05 | -2.1E-04 |
| fomepizole | -0.04 | 0.04 | -1.7E-04 |
| tolmetin sodium salt dihydrate | -0.02 | 0.02 | -9.2E-05 |
| guanabenz acetate | -0.02 | 0.02 | -8.0E-05 |
| methysergide maleate | -0.02 | 0.02 | -6.8E-05 |
| nicotine | -0.01 | 0.01 | -5.9E-05 |
| 2',3'-dideoxyinosine | -0.01 | 0.01 | -4.9E-05 |
| desipramine hydrochloride | -0.01 | 0.01 | -2.8E-05 |
